# Petrobactin, a siderophore produced by *Alteromonas*, mediates community iron acquisition in the global ocean

**DOI:** 10.1038/s41396-021-01065-y

**Published:** 2021-08-02

**Authors:** Lauren E. Manck, Jiwoon Park, Benjamin J. Tully, Alfonso M. Poire, Randelle M. Bundy, Christopher L. Dupont, Katherine A. Barbeau

**Affiliations:** 1 Geosciences Research Division, Scripps Institution of Oceanography, University of California San Diego, La Jolla, CA USA; 2 School of Oceanography, University of Washington, Seattle, WA USA; 3 Center for Dark Energy Biosphere Investigations, University of Southern California, Los Angeles, CA USA; 4 Department of Environment and Sustainability, J. Craig Venter Institute, La Jolla, CA USA; 5 Department of Human Health, J. Craig Venter Institute, La Jolla, CA USA; 6 Department of Synthetic Biology, J. Craig Venter Institute, La Jolla, CA USA

**Keywords:** Biogeochemistry, Marine microbiology

## Abstract

It is now widely accepted that siderophores play a role in marine iron biogeochemical cycling. However, the mechanisms by which siderophores affect the availability of iron from specific sources and the resulting significance of these processes on iron biogeochemical cycling as a whole have remained largely untested. In this study, we develop a model system for testing the effects of siderophore production on iron bioavailability using the marine copiotroph *Alteromonas macleodii* ATCC 27126. Through the generation of the knockout cell line Δ*asbB*::km^r^, which lacks siderophore biosynthetic capabilities, we demonstrate that the production of the siderophore petrobactin enables the acquisition of iron from mineral sources and weaker iron-ligand complexes. Notably, the utilization of lithogenic iron, such as that from atmospheric dust, indicates a significant role for siderophores in the incorporation of new iron into marine systems. We have also detected petrobactin, a photoreactive siderophore, directly from seawater in the mid-latitudes of the North Pacific and have identified the biosynthetic pathway for petrobactin in bacterial metagenome-assembled genomes widely distributed across the global ocean. Together, these results improve our mechanistic understanding of the role of siderophore production in iron biogeochemical cycling in the marine environment wherein iron speciation, bioavailability, and residence time can be directly influenced by microbial activities.

## Introduction

Iron (Fe) is an essential micronutrient for marine microorganisms, serving as a cofactor in enzymes facilitating fundamental processes such as photosynthesis, respiration, and nitrogen fixation. However, in oxygenated seawater at pH ~8, inorganic dissolved iron is most thermodynamically stable in the form of Fe(III) hydroxide complexes. These hydroxide complexes have the tendency to be scavenged by sinking particulate matter and are in equilibrium with Fe(III) oxyhydroxide particulates which are characterized by low solubility [1]. Coupled with enhanced biological uptake in the surface ocean, this results in extremely low dissolved iron concentrations in most regions of the global ocean and limits primary production by photoautotrophs in more than one-third of the surface ocean [2].

With a significant iron requirement [3–5], marine heterotrophic bacteria are also impacted by this iron scarcity and have therefore developed multiple pathways for acquiring sufficient iron from their environment [6]. One such pathway is through the production, exudation, and uptake of siderophores. Siderophores are low-molecular-weight compounds (500–1500 Da) secreted from a cell that binds strongly to iron and is subsequently acquired through outer membrane TonB dependent transporters (TBDTs) in Gram-negative bacteria. The capacity for siderophore biosynthesis by cultivated marine bacteria has been recognized now for several decades [7–14], and suites of siderophores with various chemical moieties have been isolated from cultured marine representatives [15]. The increasing availability of marine bacterial genomes and metagenomes has allowed for a more comprehensive view of the capacity for siderophore production and uptake by marine bacteria [6]. In addition, it has been demonstrated that eukaryotic phytoplankton also have the ability to utilize siderophore-bound iron [4, 16–18]. Electrochemical measurements have widely detected strong iron-binding ligands in seawater, and a portion of this strong ligand pool is likely comprised of siderophores. More recently, several structural classes of siderophores have been isolated directly from seawater [19–22].

Given these observations, it is now widely accepted that siderophores play a role in iron acquisition and cycling in the marine environment. However, despite recent advances in our understanding of the production and distribution of siderophores in the marine environment, a gap has remained in our ability to experimentally test hypotheses regarding the functional role of siderophores in the marine biogeochemical iron cycle. Siderophores comprise one fraction of a diverse pool of organic ligands that bind over 99% of the dissolved iron in seawater and largely control the bioavailability and residence time of dissolved iron in the oceans [23]. Due to their high binding affinities for iron, siderophores have the potential to capture iron from many sources and sequester it in very specific forms, including the ability to solubilize particulate forms of iron [24–29]. However, the various iron sources from which siderophores can effectively obtain iron to support bacterial growth in the marine environment have not been experimentally determined, leaving open questions regarding their role in marine microbial iron acquisition as well as their influence on overall iron cycling. Accomplishing this will be an important step in understanding and modeling the global iron biogeochemical cycle.

In recent work, the marine copiotroph *Alteromonas macleodii* ATCC 27126 has been characterized as a siderophore producer [30, 31], and a suite of putative iron transporters in this strain has been identified by transcriptomic analysis of iron-limited cultures [31]. The genus *Alteromonas* is prevalent in oceanic waters [32], and members have been found to become highly abundant in environments enriched in organic matter and nutrients [33–37]. In addition, in the iron-limited waters of the Southern Ocean, members of *Alteromonadales* displayed the highest expression of genes related to iron metabolism on a per-cell basis while comprising only 1–2% of the total prokaryotic population [38]. These previous studies suggest that this genus has the potential to disproportionately affect the processing of organic matter and its associated macro- and micronutrients [39]. In the work presented here, we aim to utilize the marine copiotroph *A. macleodii* ATCC 27126 as an environmentally relevant model organism in order to provide an improved framework, based on experimental evidence, for the role of siderophore production in iron acquisition and biogeochemical cycling in the marine environment. We accomplish this first by comparing the growth of wild-type (WT) *A. macleodii* ATCC 27126 to a generated knockout mutant, Δ*asbB*::km^r^ which lacks siderophore biosynthetic capabilities, on multiple sources of iron in culture. We then complement these culture studies with analytical and genomic field measurements in order to investigate the prevalence of petrobactin biosynthesis, the siderophore specifically produced by *A. macleodii* ATCC27126, across the global ocean.

## Material and methods

### Bacterial strains and growth conditions

*A. macleodii* ATCC 27126 WT and a generated Δ*asbB*::km^r^ line were used for all growth experiments. See below for details on the generation of the Δ*asbB*::km^r^ line. *Escherichia coli* Epi300 was used for conjugative plasmid delivery. *A. macleodii* was maintained on Marine Broth (MB) 2216 (BD Difco, Sparks, MD, USA) agar plates and liquid media at room temperature. *E. coli* was maintained on LB (BD Difco) agar plates and liquid media at 37 °C. Growth experiments as described below were conducted in PC + media prepared in artificial seawater [31, 40]. Artificial seawater was utilized in order to reduce the concentration of background dissolved organic matter and the potential for competing for iron-binding ligands in solution. See the Supplemental Materials and Methods for the complete preparation of PC + media. Media was prepared and cultures were grown using appropriate aseptic techniques.

### Partial deletion and insertional inactivation of MASE_09715

The *A. macleodii* ATCC 27126 Δ*asbB*::km^r^ line was engineered via homologous recombination [41], targeting the disruption of MASE_09715 via the insertion of a kanamycin resistance cassette. The suicide plasmid pLEM01 was generated through a four-piece Gibson assembly [42] using a Gibson assembly master kit (New England Biolabs, Ipswich, MA, USA) following the manufacture’s protocol (Table S1). The *Bacillus subtilis sacB* gene encoding levansucrase was included on the suicide plasmid opposite the homology arms and kanamycin resistance cassette (Fig. S1a), allowing for the selection of exconjugates with a double crossover at the insertion site. All polymerase chain reactions (PCR) for the generation of Gibson fragments were carried out using PrimeStar Max DNA polymerase (Takara Bio, Kusatsu, Japan). The desired assembly of pLEM01 was confirmed via PCR (Table S2, Fig. S1b). pLEM01 was transformed into chemically competent *E. coli* Epi300 cells containing the mobilization plasmid pTA-Mob followed by selection on LB plates with 50 μg/mL kanamycin. *E. coli* Epi300 containing both the pTA-Mob and pLEM01 plasmids was then mated overnight with *A. macleodii* ATCC 27126 WT on MB agar plates at 30 °C. Double crossover mutants were selected for through dilution plating onto MB plates with 50 μg/mL kanamycin and 5% sucrose. The high salinity of MB selected against the growth of *E. coli* during dilution plating. In addition, X-gal (Roche, Basel, Switzerland) was utilized at 260 μg/mL in order to visually distinguish *A. macleodii* from potentially contaminating *E. coli* colonies where the natural β-galactosidase activity of *A. macleodii* resulted in blue-stained cells. Individual exconjugant *A. macleodii* colonies were screened for the knockout phenotype on CAS plates [43] and positive phenotype colonies were further screened with PCR to confirm correct insertion (Table S2, Figure S2). The desired phenotype was also confirmed via analysis by liquid chromatography coupled to electrospray ionization mass spectrometry (LC–ESI–MS) of the culture supernatant from the mutant strain grown under iron limitation as described below.

### Siderophore characterization

Siderophore production by ATCC 27126 was assessed by solid-phase extraction (SPE) of the siderophore from iron-limited ATCC 27126 culture supernatant followed by analyses via LC–ESI–MS. Both the ATCC 27126 WT and Δ*asbB*::km^r^ cell lines were grown in PC + media without any added iron source in order to induce iron limitation. After 8 hours of growth, cultures were terminated, and the culture supernatant was collected following filtration through a 0.2 μm polycarbonate filter (Whatman, Maidstone, United Kingdom). The filtered supernatant was stored at −20 °C in the dark until analysis. See the Supplemental Materials and Methods for a detailed description of SPE and LC–ESI–MS conditions.

### Growth experiments with various iron sources common to the marine environment

*A. macleodii* ATCC 27126 WT and Δ*asbB*::km^r^ were maintained on MB 2216 agar and triplicate colonies of each were inoculated in 5 mL of liquid MB 2216 to initiate growth experiments. All liquid cultures were maintained in the dark at room temperature with shaking at 190 rpm. After 8 h of growth, 100 μL of each liquid MB culture was then transferred to 5 mL of PC + media without any added iron source. The triplicate cultures were allowed to grow for 12 h in order to reach an iron-limited state. Finally, 100 μL of each culture was then transferred to two 5 mL aliquots of fresh PC + media, one with no added iron source (iron deplete control) and the second with an added iron source at a final total iron concentration of 5 μM, unless otherwise noted. Growth was tested on mineral-based iron sources (ferrihydrite colloids and Arizona Test Dust [ATD]), discrete iron–ligand complexes (ferrioxamine B, ferric citrate, heme, and cytochrome *c*), and heterogeneous sources of organically bound iron (Suwanee River Humic Acid [SRHA] reference material and phytoplankton lysate). Due to limited substrate availability, SRHA is the only substrate where the iron concentration was added at less than 5 μM and concentrations varied across multiple SRHA treatments. See the Supplemental Materials and Methods for the specific preparation of each iron substrate tested. Cultures grown on 5 μM FeCl_3_ were used as iron-replete controls. Growth in each culture was monitored with optical density measurements at 600 nm (OD600) on a Spectronic 20 Genesys UV–VIS spectrophotometer. Maximum specific growth rates (μ max) and carrying capacity (*K*) were calculated using the growth rates package in R [44] and compared with an independent, two-tailed Student’s *t* test (*p* < 0.05).

### Field sampling and processing

Samples were collected for in situ siderophore characterization throughout the Gradients 1.0 cruise on the R/V *Ka’imikai-O-Kanaloa*, between April 19 and May 4, 2016 at 8 stations along 158°W, between 23.5°N and 37.3°N. Ten to twenty liters of seawater were collected from discrete depths between the surface and 400 m with a trace metal clean rosette outfitted with Teflon-coated external spring Niskin bottles (Ocean Test Equipment, Fort Lauderdale, FL, USA) on a non-metallic line. Seawater was filtered through a 0.2 μm Acropak 200 capsule filter (Pall, Port Washington, NY, USA) and then pumped at a flow rate of 18 mL/min onto a Bond Elut ENV column (6 mL, 1 g resin) (Agilent Technologies, Santa Clara, CA, USA). The column was rinsed with two column volumes of Mili-Q water and stored at −20 °C until analysis via LC–ESI–MS. See the Supplemental Materials and Methods for a detailed description of SPE and LC–ESI–MS conditions.

### Detecting petrobactin-like biosynthetic pathways in marine bacterial genomes

**Fig. 1:**
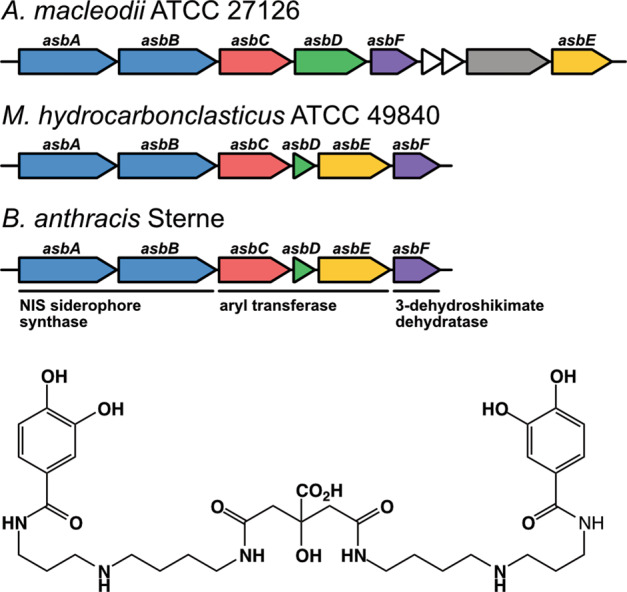
The biosynthetic gene clusters of the known petrobactin producers *A. macleodii* ATCC 27126, *M. hydrocarbonclasticus* ATCC 49840, and *B. anthracis* Sterne are depicted with the petrobactin structure below. Genes are colored and labeled according to sequence similarity with the well-characterized *B. anthracis* biosynthetic gene cluster. A PepSY domain-containing gene (gray) and genes encoding two hypothetical proteins (white) are also present within the *A. macleodii* ATCC 27126 biosynthetic gene cluster.

A search for homologous biosynthetic gene clusters for petrobactin in uncultivated prokaryotes was conducted with the collection of metagenome-assembled genomes (MAGs) from the *Tara* Oceans dataset [45]. Search strategies aimed to identify the co-location of genes encoding non-ribosomal peptide synthase independent (NIS) siderophore synthases (*asbAB*) with those encoding 3-dehydroshikimate dehydratase (*asbF*). Together, these genes are diagnostic of biosynthetic pathways for petrobactin or petrobactin-derivatives [46–49] (Fig. 1). MAG assemblies were downloaded from NCBI (PRJNA391943) and protein-coding genes were detected with Prodigal (v2.6.3) [50]. The hidden Markov models for the conserved domains found within *asbAB* and *asbF* (pfam04183 and pfam01261, respectively) were searched against the detected protein-coding genes for each assembly using HMMER (v3.3) [51]. For assemblies where a positive hit for both pfam domains was detected, the co-localization of the two domains within the genome was manually determined. Pfam domains were then assigned to neighboring genes in order to determine the presence of a complete biosynthetic pathway.

The entire *Tara* Oceans metagenome dataset (reads representing 238 samples) was then competitively mapped to the identified MAGs with complete petrobactin-like biosynthesis pathways using Bowtie2 (v2.3.4.1) [52]. Results were filtered with BamM (v1.7.0) to remove reads that were less than 95% identical and had less than 75% alignment. Results were then converted to read count per contig using featureCounts (v1.5.3) [53] as implemented within BinSanity [54]. Read counts for individual MAGs were then normalized to reads per kbp genome per Gbp of metagenome (RPKM) for the filtered recruitment per sample. Identified MAGs that shared greater than 95% average nucleotide identity (ANI), as determined using FastANI (v1.3) [55] with a fragment length of 1500 bp, were considered to be members of the same species. As MAG assembly is semi-stochastic, MAGs of the same species will contain overlapping segments as well as unique segments. During competitive read recruitment, overlapping segments would split reads among MAGs of the same species while unique segments would not. In order to account for this, we aimed to evaluate the genome coverage of MAGS represented by a species-level environmental population in order to capture a comprehensive view of the distribution of the petrobactin-like biosynthetic gene cluster. To achieve this, the read counts per contig calculated above from a given sample for all MAGs of the same species were summed. To calculate an approximate RPKM value for the combined read counts, the length of the average complete species genome was estimated by taking the average of the length of each MAG multiplied by the corresponding percent completeness of that MAG, as calculated with CheckM [56].

## Results

### Petrobactin production by *A. macleodii* increases the bioavailability of specific iron sources

Previous work has identified a putative NIS-type siderophore biosynthesis gene cluster in ATCC 27126 [30, 31] which was significantly upregulated under iron limitation [31]. Siderophore production was confirmed with a CAS assay [30, 31] and putatively identified as petrobactin based on sequence similarity of the biosynthetic gene cluster in ATCC 27126 to the characterized *asbABCDEF* operon in *Bacillus anthracis* [30] (Fig. 1). In the current work, LC-ESI-MS analysis of culture supernatant from iron-limited *A. macleodii* ATCC 27126 confirmed the siderophore produced by the WT strain as petrobactin. An MS^1^ peak at 719.361 *m/z* was detected, corresponding to the expected [M + H]^+^ *m/z* value for petrobactin (C_34_H_50_N_6_O_11_) from a comprehensive list of known siderophore structures [57] (Fig. S3a). The MS^2^ spectra collected from the MS^1^ peak at 719.361 *m/z* further confirmed this identification with fragmentation peaks at 194, 282, 438, and 565 *m/z* correspondings to previously reported MS^2^ spectra for petrobactin [47, 58] (Fig. S3c).

The *A. macleodii* ATCC 27126 Δ*asbB*::km^r^ cell line was engineered via the insertional inactivation of MASE_09175 (putative *asbB* within the biosynthetic gene cluster of ATCC 27126, Fig. 1) with a kanamycin resistance cassette (Fig. S2). The disruption of MASE_09175 completely eliminated the production of petrobactin in the Δ*asbB*::km^r^ cell line as determined with a CAS assay (Fig. S4) and LC–ESI–MS analysis of culture supernatant from iron-limited cells (Fig. S3b).

**Fig. 2:**
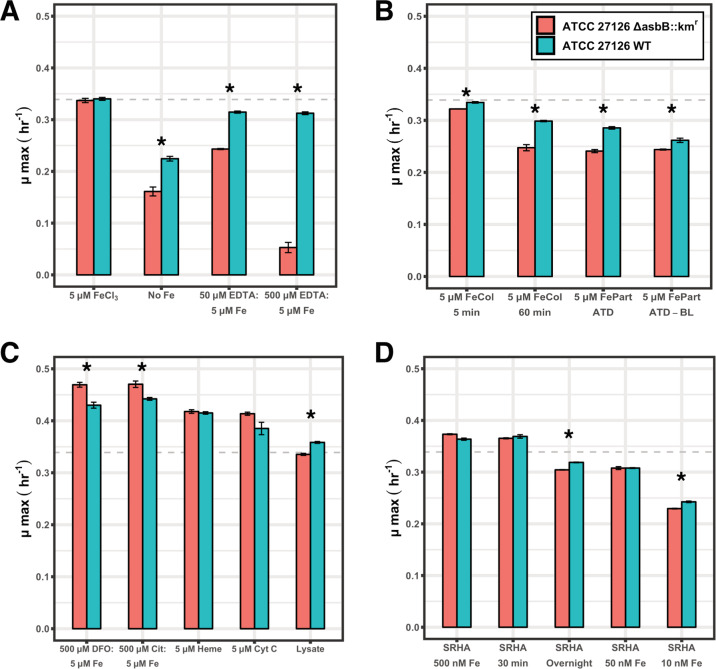
Maximum specific growth rates (μ max) of the *A. macleodii* ATCC 27126 WT and Δ*asbB*::km^r^ strains on tested iron sources. Conditions marked with an asterisk indicate a statistically significant difference between the WT and Δ*asbB*::km^r^ strains (independent, two-tailed *t* test, *p* < 0.05). Error bars represent the standard deviation of measurements from biological triplicates. A horizontal dashed line is a reference point for the observed growth rate of *A. macleodii* ATCC 27126 on FeCl_3_ as an iron source. **A** Iron controls and EDTA treatments. **B** Colloidal and particulate mineral iron sources, 5 μM FeCol (5 min): Fe colloids synthesized with 5 min of heating and added at a total iron concentration of 5 μM, 5 μM FeCol (60 min): Fe colloids synthesized with 60 min of heating and added at a total iron concentration of 5 μM, 5 μM FePart ATD: particulate iron added as Arizona Test Dust at a total iron concentration of 5 μM, 5 μM FePart ATD-BL: particulate iron added as Berger-leached Arizona Test Dust at a total iron concentration of 5 μM. **C** Biogenic sources of chelated iron each added at a total iron concentration of 5 μM, DFO desferrioxamine B, Cit citrate, Cyt c cytochrome *c*, Lysate *T. pseudonana* lysate added as the sole iron source. **D** SRHA treatments, SRHA 500 nM Fe: unmodified SRHA added at a final iron concentration of 500 nM, SRHA 30 min: SRHA extracted for 30 min with AG50W-X8 cation exchange resin and added at the same volume as unmodified SRHA to final cultures, SRHA Overnight: SRHA extracted overnight with AG50W-X8 cation exchange resin and added at the same volume as unmodified SRHA to final cultures, SRHA 50 nM Fe: unmodified SRHA added at a final iron concentration of 50 nM, SRHA 10 nM Fe: unmodified SRHA added at a final iron concentration of 10 nM.

The growth of the *A. macleodii* ATCC 27126 WT strain was then compared to that of the Δ*asbB*::km^r^ mutant strain on a range of iron sources common to the marine environment in order to determine the effects of siderophore production on iron bioavailability. Ferrihydrite colloids and ATD were utilized as representatives of lithogenic iron sources to the marine environment. EDTA-bound iron and SRHA reference material served as model substrates for weakly complexed iron–ligand pools. Ferrioxamine B, ferric citrate, heme, cytochrome *c* (a heme protein with a covalently bound heme prosthetic group), and phytoplankton lysate represented dominant sources of biogenically complexed iron. With the exception of the SRHA treatments, all iron sources were added at a final total iron concentration of 5 μM. The results of these experiments (growth curves and calculated growth rates and culture carrying capacities) are displayed in Figs. 2, S5, S6, and S7. Growth on 5 μM FeCl_3_ was used as a control for replete iron conditions, and both the WT and Δ*asbB*::km^r^ strains grew equally well under these conditions (Figs. 2a, S5a, and S7a). Cultures without the addition of any iron source were used as controls for iron deplete conditions.

In order to test the role that petrobactin production plays in the acquisition of iron from colloidal and particulate minerals, the growth of the WT and Δ*asbB*::km^r^ strains was compared on ferrihydrite colloids synthesized with both 5 and 60 min of heating, the latter of which is less labile [59]. Growth was also tested on ATD in both its unmodified form as well as following a Berger leach, again the latter of which is expected to contain less labile iron. ATD is a commercially available mineral dust material that is well characterized for its trace metal content and is being assessed as a consensus reference material for aerosol studies [60, 61]. Operationally, the Berger leach is utilized to remove the bioavailable fraction of iron from particulates via a reducing weak acid leach [62]. The total iron concentration of each of these treatments in culture was held constant at 5 μM. The WT strain grew at a faster rate and achieved higher cell densities compared to that of the Δ*asbB*::km^r^ strain on each of these mineral iron sources (Figs. 2b, S5b, and S7b). When grown on ferrihydrite colloids synthesized under 5 min of heating, the maximum specific growth rate of the Δ*asbB*::km^r^ strain was only slightly less than that of the WT strain which remained nearly equal to growth on the FeCl_3_ control (Fig. 2b). When grown on either ferrihydrite colloids synthesized under 60 min of heating or ATD, the growth of both strains decreased compared to the FeCl_3_ control indicating there was a fraction of iron inaccessible to both strains from these iron substrates. However, the maximum specific growth rate and maximum cell density of the Δ*asbB*::km^r^ strain were below those of the WT, with the Δ*asbB*::km^r^ strain reaching only 56% of the maximum cell density of the WT strain when grown on ATD (Fig. S7b). Similar results were obtained for the Berger-leached ATD, the least labile of all mineral sources tested. While there was an observed decrease in the growth rates for both the Δ*asbB*::km^r^ and WT strains on this iron source compared to unmodified ATD, the Δ*asbB*::km^r^ strain again reached only ~60% of the maximum cell density of the WT strain (Fig. S7b). Together, these results indicate that petrobactin solubilizes a portion of total iron from colloidal and particulate sources for biological uptake.

In additional experiments, EDTA was used as a weak ligand in order to control the amount of free iron (Fe′) in the solution. The total iron concentration in the media was held constant at 5 μM, but varying concentrations of EDTA resulted in treatments with ~5 and 0.5 nM Fe′, leaving the majority of iron in the form of a Fe–EDTA complex (Supplemental Materials and Methods). The WT strain was able to maintain maximum specific growth rates under each of these experimental conditions nearly equal to that of growth on the FeCl_3_ control (Fig. 2a). In contrast, the growth of the Δ*asbB*::km^r^ strain responded strongly to the Fe′ concentration, and with less than 1 nM Fe′ in solution, the maximum specific growth rate and maximum cell density of the Δ*asbB*::km^r^ strain drastically decreased, falling even below that of the iron deplete control (Figs. 2a, S5a, and S7a). These results indicate that the WT strain was able to access iron bound to EDTA, thereby accessing the total iron in solution via the production of petrobactin, while the Δ*asbB*::km^r^ strain was reliant solely on Fe′ as an iron source.

In contrast, when grown on the discrete organic iron–ligand complexes ferrioxamine B, ferric citrate, heme, and cytochrome *c*, both the WT and Δ*asbB*::km^r^ strains exhibited similar growth rates and maximum cell densities with growth rates actually exceeding those of growth on FeCl_3_ (Figs. 2c, S5c, and S7c). In addition, the growth rates of the Δ*asbB*::km^r^ strain were statistically greater than those of the WT when grown on ferrioxamine and ferric citrate complexes. However, both strains reached the same maximum cell densities on these sources indicating that ultimately the same total amount of iron from these sources was accessible to both strains. Based on the preparation of these substrates, the concentrations of Fe′ in these experiments are expected to be negligible (Supplemental Materials and Methods). Therefore, growth can be interpreted as the ability to acquire iron from the iron-ligand complex itself and reinforces the ability of *Alteromonas* spp. to efficiently utilize larger, organic substrates [31].

Growth of the WT and Δ*asbB*::km^r^ strains was also tested on heterogeneous sources of organically complexed iron. This included unfiltered phytoplankton lysate (therefore comprised of heterogenous soluble, colloidal, and particulate organic iron phases) as well as SRHA reference material, consisting of dissolved iron associated with humic acids. The maximum specific growth rate of the Δ*asbB*::km^r^ strain on phytoplankton lysate as an iron source was slightly lower compared to that of the WT strain (Fig. 2c), indicating that a fraction of iron was made readily available by siderophore production allowing for more efficient growth. However, maximum cell densities of Δ*asbB*::km^r^ equal to those of the WT strain were achieved (Figs. S5d and S7c) indicating that ultimately a similar magnitude of bioavailable iron was present in the lysate regardless of siderophore production. When grown on SRHA, differences in growth were only observed once the most labile fractions of iron were removed or diluted below replete levels (Supplemental Materials and Methods). In these treatments, the WT reached somewhat higher growth rates and maximum cell densities compared to the Δ*asbB*::km^r^ strain (Figs. 2d, S6a, S6b, and S7d) indicating that a small fraction of iron associated with SRHA seems to be available to the WT strain specifically through the production of petrobactin but that a large fraction is highly bioavailable even in the absence of siderophore production.

### Detection of petrobactin in the North Pacific

**Fig. 3:**
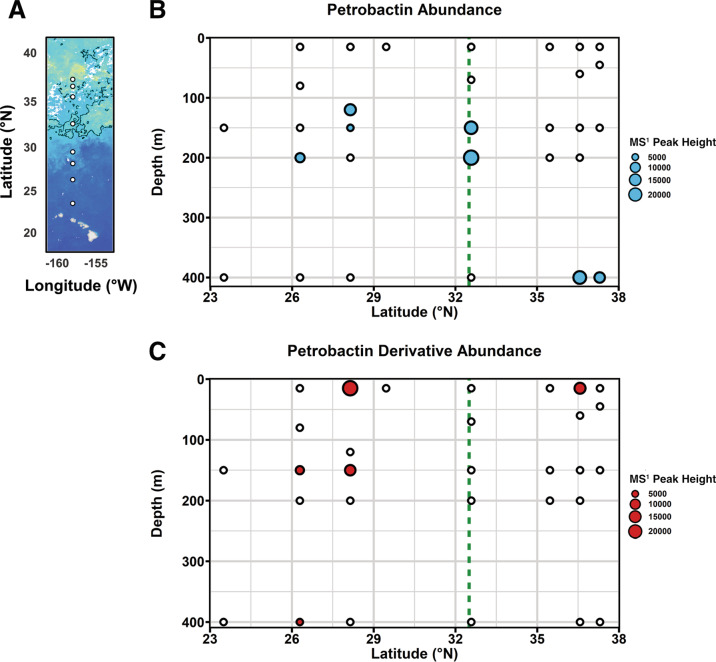
Distribution of petrobactin across the North Pacific as detected during April 2016. **A** Station map of siderophore sampling locations along 158°W between 23 and 38°N collected during the Gradients 1.0 cruise overlain on MODIS sea surface chlorophyll concentrations (mg m^−^^3^, 4  km resolution, monthly averages for April 2016). Black lines depict the 0.2 mg m^−3^ chlorophyll contours. **B** Distribution of petrobactin (C_34_H_50_N_6_O_11_) along this transect. Petrobactin was detected in samples depicted with filled blue circles and the size of the circle is proportional to petrobactin abundance as determined by the MS^1^ peak height. Petrobactin was not detected in samples represented by open circles. The dashed line marks the TZCF. **C** Distribution of a petrobactin derivative (C_32_H_46_N_6_O_11_) along this transect. The petrobactin derivative was detected in samples depicted with filled red circles and the size of the circle is proportional to the derivative abundance as determined by MS^1^ peak height. The derivative was not detected in samples represented by open circles. The dashed line marks the TZCF.

The Gradients 1.0 cruise along 158°W in the North Pacific sampled across a wide range of biogeochemical conditions moving from the North Pacific Subtropical Gyre into the North Pacific Transition Zone (Figs. 3a and S8). The transition zone chlorophyll front (TZCF; defined as the 0.2 mg m^−3^ surface chlorophyll contour [63]) was located at 32.5°N (Fig. 3a). Surface nitrate (NO_3_^−^) concentrations remained close to detection limits until crossing the TZCF where concentrations significantly increased and surface maximums near 6 μM were observed (Fig. S8c) [64]. Dissolved iron concentrations at the surface remained below 0.5 nM across the entire transect, reaching minimum values near 0.06 nM across the TZCF (Fig. S8d). MS^1^ peaks corresponding to petrobactin (719.361 *m/z* [M + H]^+^, C_34_H_50_N_6_O_11_) and a putative petrobactin derivative (691.330 *m/z* [M + H]^+^, C_32_H_46_N_6_O_11_) were detected in the extracts of seawater samples collected from this transect following LC–ESI–MS analysis (Fig. 3b, c). Both of these siderophores were detected exclusively in the apo form; however, it is unclear at this point if this is a true environmental phenomenon or a result of sample processing. Petrobactin was detected exclusively in samples collected from below 100 m, primarily between 100 and 200 m (Fig. 3b). However, beyond the TZCF petrobactin was detected exclusively at 400 m. In contrast, the petrobactin derivative was detected primarily between the surface and 150 m across the entire transect but was detected as deep as 400 m in a single sample (Fig. 3c). Petrobactin was detected in samples with a range of dissolved iron from 0.2 to 1.0 nM.

### Distribution of petrobactin-like biosynthetic pathways in the marine environment

A search for petrobactin biosynthetic pathways within MAGs assembled from the *Tara* Oceans dataset [45] detected 66 genomes encoding at least one putative NIS-type siderophore synthase. Of these 66 positive hits, five genomes exhibited the co-localization of genes encoding the NIS-type synthase with a putative 3-dehydroshikimate dehydratase (*asbF*). Manual inspection confirmed a complete petrobactin-like biosynthetic pathway in each of these five MAGs (Fig. S9). Putative TBDTs and ATP-binding cassette transporters for iron substrates adjacent to these biosynthetic gene clusters further support this annotation. Four of these genomes are classified as belonging to the *Marinobacter* genus (SP36, IN15, EAC19, and ARS1015) and share >96% ANI with each other, indicating they are members of the same species. The fifth MAG is an unclassified *Alteromonadaceae* (EAC69). A similar search for petrobactin-like biosynthetic pathways in the genomes of cultured bacterial isolates revealed a more diverse distribution of this pathway across *Gammaproteobacteria*, *Alphaproteobacteria*, and *Bacilli* (Supplemental Results, Fig. S10, Dataset S1).

**Fig. 4:**
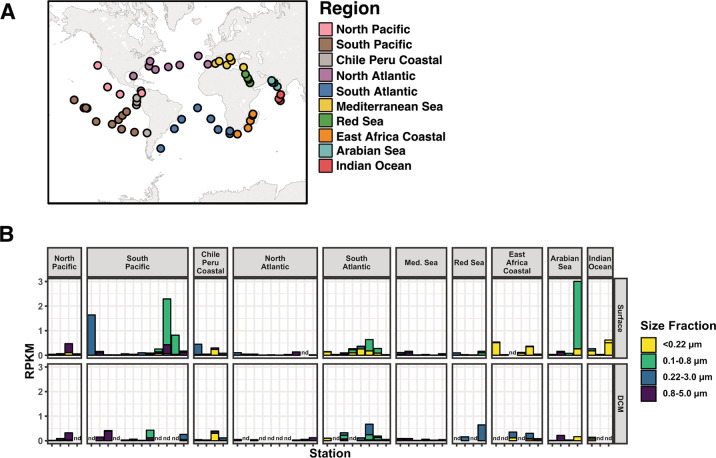
Distribution of total reads from the Tara Oceans dataset that recruited to four Marinobacter MAGs sharing >95% ANI and containing a petrobactin-like biosynthetic gene cluster. **A** Station map of *Tara* Oceans sampling locations colored according to region. **B** Abundance of reads that recruited to the *Marinobacter* MAGs represented as a species-level environmental population at both the surface (top panel) and DCM (bottom panel) across the *Tara* Oceans dataset. RPKM values were determined by summing the reads recruited to all four MAGs belonging to the species for a given sample and using an estimated complete species genome length for normalization (see Materials and Methods for complete details). Samples grouped according to the oceanic region as defined in panel A and colored by corresponding sample size fraction. Columns labeled nd indicates that a sample was not collected at that depth for a given station. Raw data for this figure can be found in Dataset S2.

When reads from the entire *Tara* Oceans metagenomic dataset [65, 66] are mapped to the five MAGs with complete petrobactin-like biosynthetic pathways, they are detected in every major ocean basin sampled by the *Tara* expedition at both the surface and deep chlorophyll maximum (DCM) (Figs. 4, S11, and S12). Due to the high ANI between the four *Marinobacter* genomes, a combined read count was constructed in order to capture the full breadth of the species-level environmental population across the global ocean (see Materials and Methods for calculation details) (Fig. 4, Dataset S2). At this level of analysis, the highest read counts from the *Marinobacter* MAGs were detected at the surface in the South Pacific and Arabian Sea (Fig. 4). Read counts attributed to the individual *Marinobacter* MAGs all follow a similar global distribution with variations capturing more specific phylogenetic relationships (approximately equivalent to strains) such that reads associated with ARS1015 were detected in the North Atlantic and the Mediterranean Sea at a greater abundance compared to the other three *Marinobacter* MAGs (Fig. S11b). At the DCM, distributions of the *Marinobacter* genomes followed a similar pattern to the surface (Figs. 4 and S12b). Read counts for the *Alteromondaceae* MAG (EAC69) were generally lower than those of the *Marinobacter* MAGs by 1–2 orders of magnitude (Figure S11c, S12c). However, in a single sample from the East Africa Coastal region the genome coverage of EAC69 was greater than that of the *Marinobacter* MAGs. Read counts for EAC69 were found at a similar magnitude in surface samples and at the DCM with the exception of the South Pacific where read counts were up to an order of magnitude greater at the DCM than in corresponding surface samples (Fig. S12c).

## Discussion

It is now recognized that a significant fraction of dissolved iron in the marine environment is complexed by a pool of organic ligands, allowing dissolved iron to accumulate to concentrations above that set by its inorganic solubility [23]. A growing number of measurements of iron-binding ligands in the marine environment in recent years, both through electrochemical techniques and the direct isolation of ligands such as siderophores, reveal a dynamic ligand pool [20–23, 67]. In addition, model studies have concluded that ligand concentrations have a large influence on iron availability and resultant global carbon cycling, even more so than variations in direct iron input [68]. Yet, while organic ligands have emerged as major controls on iron biogeochemical cycling, much remains to be learned regarding their sources, sinks, and definitive roles in the marine environment. As a significant fraction of the total iron-binding ligand pool, understanding the impact of siderophores on iron biogeochemical cycling will be an important step in this process. We have demonstrated that the production of the siderophore petrobactin by a marine copiotrophic bacterium increases the bioavailability of iron from a number of discrete iron sources. Furthermore, petrobactin has been detected for the first time directly from seawater in the North Pacific, and genomic evidence points to a wide distribution of the petrobactin biosynthetic pathway in the marine environment. As discussed below, these results shed light on the role of siderophore production for iron acquisition by marine microbes and the resultant implications of siderophore biosynthesis for iron biogeochemical cycling.

### A significant role for siderophore production in the incorporation of new iron into the marine environment

**Fig. 5:**
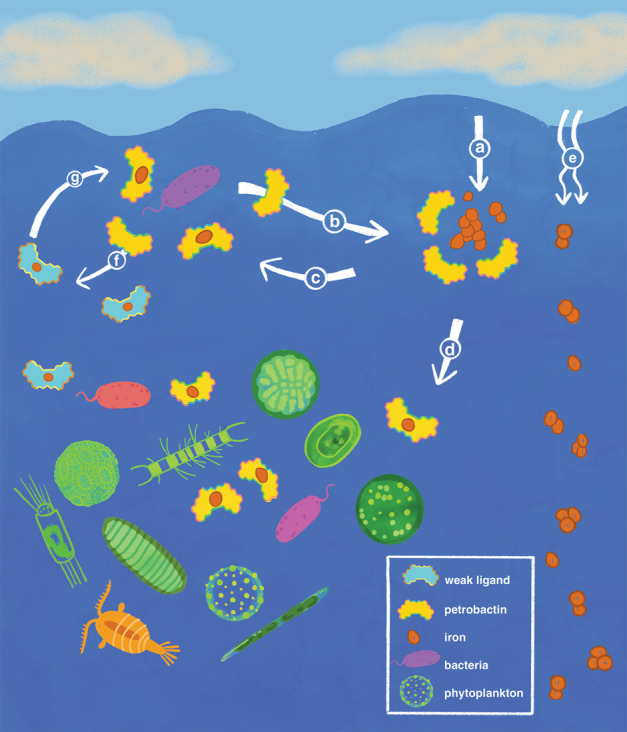
Proposed role of petrobactin production on the bioavailability of iron in the marine environment. **a** Iron in the form of lithogenic particles is delivered to the surface ocean via atmospheric dust. **b** Petrobactin is produced and released into the marine environment by *A. macleodii* and additional petrobactin-producing marine bacteria. **c** Petrobactin binds iron from lithogenic particles transferring this iron to the dissolved iron pool where it can be acquired by the producing bacterium. **d** The dissolved iron-petrobactin complex is also now available to the greater microbial community where this iron may be acquired via one of several potential mechanisms such as the direct acquisition of the complex, reductive dissociation as Fe(II), or exchange with another strong ligand followed by subsequent transport. **e** In the absence of petrobactin, iron remains within the lithogenic particulate pool and sinks out of the surface ocean in a largely non-bioavailable form. **f** Petrobactin also interacts with iron bound to weaker organic ligands in the marine environment. **g** Petrobactin acquires iron from weaker ligands via ligand exchange converting it to a form readily taken up by *A. macleodii* and other microbes with appropriate transport mechanisms. Illustration credit: Rachael Chan, The Art Center College of Design.

The major sources of new iron to the marine environment include atmospheric dust, hydrothermal vents, and the resuspension of sediments along continental margins [67]. Iron from these sources largely consists of colloidal and particulate mineral phases that are characterized by low solubility in oxygenated seawater. Given the tendency of these particles to sink and be scavenged from the water column, it is unclear how much iron from these particulate sources ultimately contributes to the dissolved and bioavailable pools of iron in the marine environment. The role of siderophore production in the acquisition of iron from these mineral sources has long been hypothesized. In particular, the relatively recent recognition that hydrothermal input contributes a significant fraction of new iron to the marine environment [69, 70] has been largely attributed to the organic complexation [71–73] and microbial acquisition [74] of iron from this source. While dissolution and modeling studies support these conclusions [24–29, 75], up to this point, there has been a lack of experimental evidence demonstrating that siderophore-based acquisition of iron from mineral sources supports bacterial growth in the marine environment. Through the generation of a knockout mutant and the use of a controlled biological system, we have shown that petrobactin production by a marine bacterium liberates a fraction of iron from colloidal and particulate mineral iron sources for biological use (Figs. 2b and S7b). An outstanding research question is whether this behavior is shared with other siderophores [76], but these results further suggest that siderophore production is a significant mechanism by which iron from external, particulate inputs can be incorporated into soluble and bioavailable pools of iron (Fig. 5). While directly benefitting the siderophore-producing bacterium, the solubilization of iron from particulates by siderophores also has the potential to confer benefits to non-siderophore producing microbes that can access iron from iron–siderophore complexes but otherwise would be unable to directly utilize iron from lithogenic sources [77]. Furthermore, the siderophore-driven dissolution of iron from particles and the subsequent acquisition of these iron-siderophore complexes by marine microbes transitions this mineral-phase iron to a new pool contained within biomass where its speciation is further transformed and where it may be retained for biological use on longer timescales via recycling [78]. This confirms an important role for biologically produced iron-binding ligands in controlling the balance between the scavenging and retention of dissolved iron in the marine environment [79–82]. These results place further emphasis on the need to understand factors influencing siderophore production, degradation, and distribution as these are likely some of the major driving forces controlling the amount of new iron effectively incorporated into the marine environment and available for biological use.

### Petrobactin production can effectively sequester iron from weak to intermediate iron-ligand complexes

In the results presented here, the most pronounced growth advantage observed in the WT due to siderophore production was growth on EDTA-bound iron (Figs. 2a and S7a). This demonstrates that siderophore production can increase the bioavailability of iron from exogenous iron–ligand compounds, particularly when a direct transport mechanism for the complex is absent, as would be the case for the synthetic ligand EDTA. With a relatively weak binding strength for iron in seawater [83], EDTA serves as a model for the weakest class of iron-binding ligands found in the marine environment [23]. With conditional stability constant with respect to Fe′ of 10^11.4^ [84], petrobactin would be expected to effectively sequester iron from EDTA, allowing the WT to access the total amount of iron in solution. In contrast, without petrobactin, the Δ*asbB*::km^r^ strain is dependent solely on Fe′ as an iron source in these treatments. Likewise, SRHA is a well-characterized reference material consisting of humic acids of terrestrial origin and has been characterized to have an intermediate binding strength to iron in seawater [85]. Particularly in coastal and estuarine environments, humic-like substances can play an important role in iron biogeochemistry [22, 85–89]. In these environments, it has been observed that iron-binding humics could account for the entire iron-ligand pool [85] and up to 5% of the total dissolved organic carbon pool [90]. Currently, there is no known cellular transporter that would allow marine bacteria to directly access these iron–ligand complexes, yet there appears to be a large fraction of labile iron within SRHA. However, when the most labile fractions were removed or diluted below replete levels, a growth advantage was conferred to the WT strain with the production of petrobactin (Figs. 2d and S7d). This implies that a portion of iron complexed by humic-like substances in the marine environment is accessible explicitly via exchange with a strong ligand as has been previously suggested [87]. In addition to humic-like substances, additional classes of weaker iron-binding ligands that are not structurally well-defined but are widely distributed across the global ocean include compounds excreted by cells, such as exopolymeric substances and saccharides, as well as biological degradation products [91]. It is likely that petrobactin, along with other siderophores, also plays a role in obtaining iron from this non-specific yet abundant pool of ligands (Fig. 5).

In contrast, the Δ*asbB*::km^r^ strain was able to maintain maximum growth rates equivalent to those of the WT when tested on various discrete, strong, biogenic iron-ligand complexes (Figs. 2c and S7c). In addition, both of these strains grew at significantly faster rates on these organic sources compared to the FeCl_3_ control. These results likely reflect the high transport capacity for exogenous iron–ligand complexes found within the ATCC 27126 genome. Unlike the synthetic ligand EDTA, iron-ligand complexes common to the marine environment can be acquired directly by Gram-negative bacteria via TBDTs. Previous work with this strain identified putative TBDTs for the acquisition of at least 11 different iron–ligand complexes, five of which are thought to be for the acquisition of exogenous siderophores [31]. The results presented here demonstrate that ATCC 27126 does indeed acquire organically complexed iron extremely efficiently. It is also interesting to note the ability of both strains to grow on cytochrome *c* as an iron source. Cytochrome *c* is presumed to be too large to cross the bacterial cell membrane in its entirety. In addition, the heme cofactor is covalently bound to the protein, rendering it unavailable via transport through a TBDT without further processing [40]. This suggests that either the heme molecule or the iron atom within cytochrome *c* is extracted extracellularly, requiring additional cellular machinery beyond a TBDT. However, petrobactin does not appear to play a critical role in this process. All else being equal, these results indicate that no advantage from petrobactin production is conferred in the acquisition of iron from discrete iron-ligand complexes when a corresponding transport mechanism is present. In fact, given that the Δ*asbB*::km^r^ strain could actually maintain faster growth rates on several of these organic complexes, it is possible that the investment in petrobactin production by ATCC 27126 may actually hinder growth in the presence of an iron source that can be directly taken up by the cell. Recent work with the marine copiotroph *Vibrio cyclitrophicus* 1F-53 has suggested that siderophore production can be regulated by the presence of an exogenous, bioavailable siderophore [92].

The high transport capacity of ATCC 27126 for biogenic iron-ligand compounds may also explain the similar growth observed between the WT and Δ*asbB*::km^r^ strains on phytoplankton lysate as an iron source (Figs. 2c and S7c). Cellular transporters for biogenic iron substrates such as heme or iron-containing proteins found within the lysate would allow the mutant strain to acquire sufficient iron in order to keep pace with the WT. This highlights the significant role that cellular transporters, in particular, may serve in the uptake of remineralized iron sources from phytoplankton biomass. However, for siderophore-producing marine bacteria with fewer available transporters, siderophore production may be a relatively more important strategy for acquiring biogenic iron. Alternatively, siderophore production may impart a competitive advantage in a mixed community by sequestering iron in a more selective form. The production of strong ligands presumed to be siderophores has been detected at the peak of a stimulated bloom [37], and direct measurements of ferrioxamine siderophores have been made during the degradation of sinking particles [20, 93] suggesting that siderophore production does indeed play a role in iron remineralization processes in a natural community. The detection of five TBDTs for exogenous siderophores in the genome of *A. macleodii* ATCC 27126 [31] also suggests the production of multiple types of siderophores within a microbial community is common in environments enriched in organic matter, making siderophore piracy an advantageous strategy for iron acquisition under these conditions [94]. Further work characterizing these dynamics is merited.

### The production of petrobactin, a photochemically reactive siderophore, is widespread in the marine environment

To date, siderophores isolated directly from seawater have fallen within two structural classes—either ferrioxamine—or amphibactin-type siderophores [19–22]. Ferrioxamines are hydrophilic siderophores with hydroxamate chelating groups, whereas amphibactins are characterized by a chelating polar head group bound to a fatty acid tail [15]. In contrast, petrobactin is characterized by two catecholate chelating groups joined via a citrate backbone (Fig. 1). The detection of this third structural class of siderophores across the North Pacific expands our understanding of the cycling of different classes of siderophores in the marine environment and narrows the gap between the wealth of siderophores isolated in culture and the relatively few structures detected directly in the marine environment thus far. In addition, the α-hydroxycarboxylic acid moiety in the citrate backbone of petrobactin means it is subject to photochemical decarboxylation when bound to Fe(III) [58]. Photolysis of an iron–siderophore complex results in the reduction of Fe(III) to Fe(II) and an initial loss of complexation. This change in iron speciation will inherently affect iron availability and is thought to be a significant factor in iron biogeochemical cycling within the marine environment [95]. While photochemical degradation is generally considered a loss term for strong iron-binding ligands [96], in the case of petrobactin, decarboxylation leaves much of the original structure intact, including the chelating catecholate groups, resulting in an almost unchanged binding affinity for iron [84]. The petrobactin photoproduct, therefore, represents another significant iron-binding ligand pool that may be present in the marine environment, although it was not specifically searched for in this dataset. The detection of a photoreactive siderophore directly from seawater is a significant step in understanding the cycling of this class of siderophores in the natural marine environment which has the potential to act as a unique control on iron availability [95].

While the proposed petrobactin derivative detected in this study has not been isolated from a marine bacterium to date, the biosynthesis of this derivative has been demonstrated in vitro via the petrobactin biosynthetic pathway of *B. anthracis* utilizing the starting substrate norspermidine in place of spermidine, which links the catecholate moiety to the citrate backbone of petrobactin [97]. Both norspermidine and spermidine are polyamines known to be synthesized by a range of marine microbes but with notable taxonomic differences in the prevalence of these biosynthetic capabilities [98–100]. Within *Gammaproteobacteria*, spermidine is commonly synthesized across multiple orders while norspermidine is primarily produced by members of the order *Vibrionales* [99]. Based on these taxonomic differences, the distribution of these two petrobactin structures across the North Pacific may be reflective of the ability of the corresponding microbial communities to synthesize or obtain the respective starting substrates. Alternatively, it is possible that differences in the photochemical activity of these two compounds could account for differences in their distribution. As discussed above, the photochemical activity of petrobactin is well characterized, and loss due to photodegradation could explain the absence of petrobactin in the top 100 m of the North Pacific. While the petrobactin derivative would also be expected to be subject to photochemical loss due to the presence of a citrate backbone, this has not been experimentally determined. The presence of the derivative in sunlit waters of the North Pacific may suggest different photochemical dynamics between these two compounds. Additional work will be needed to determine whether differences in microbial communities, photochemical activity, or some combination of the two are responsible for controlling the distribution of these two siderophores in the marine environment.

Beyond the direct detection of petrobactin in the North Pacific, our examination of available genomic data, from both MAGs and cultivated strains, indicates that the potential for petrobactin biosynthesis is concentrated within the classes *Alphaproteobacteria*, *Gammaproteobacteria*, and *Bacilli* (Figs. S9 and S10) but is geographically widespread in the marine environment (Figs. 4, S11, and S12). On their own, cultured members of *Alteromonas* possessing a petrobactin, biosynthetic gene cluster are some of the most prevalent species in the *Tara* Oceans dataset [32]. Our results substantiate earlier findings, based on the detection of *asbE* homologs via qPCR, which suggested that up to 2% of total prokaryotic cells in the North Atlantic may possess a petrobactin-like biosynthetic pathway [101]. The wide geographic distribution of species with the capacity to produce petrobactin or petrobactin-like derivatives implies it is playing a significant role in iron acquisition and cycling across the marine environment and renders the results presented here as ecologically significant. As is the case for many characterized siderophore-producing marine bacteria, these confirmed or putative petrobactin-producing strains are largely copiotrophic species that can quickly become dominant members of the community and exert a significant influence on biogeochemical cycling. The ability to access an otherwise non-bioavailable pool of iron, particularly that from colloidal and particulate sources, via the production of petrobactin may be an important factor in this copiotrophic lifestyle.

## Conclusion

In summary, we have utilized *A. macleodii* ATCC 27126 as an effective model organism in order to demonstrate experimentally that the acquisition of iron from specific sources in the marine environment is facilitated by the production of a siderophore, in this case, petrobactin. In particular, the bioavailability of colloidal and particulate mineral iron and iron associated with weaker ligand pools was increased through the production of petrobactin. This demonstrates the significant role that heterotrophic bacteria play in the incorporation of new iron into the marine environment via siderophore production (Fig. 5). We also report the first detection of petrobactin, a photochemically reactive siderophore, directly from seawater and have found, based on genomic evidence, that the capacity for petrobactin production is widespread throughout the marine environment. Together these results advance our mechanistic understanding of the role of siderophore production in the marine environment providing an improved framework for understanding iron biogeochemical cycling wherein iron speciation, bioavailability, and residence time can be directly influenced by microbial activity.

## Supplementary information

Supplemental Material

Dataset S1

Dataset S2
