# Supplementary material for: Petrobactin, a siderophore produced by Alteromonas, mediates community iron acquisition in the global ocean
Source: ISME J. 2021 Aug 2;16(2):358–69. doi: 10.1038/s41396-021-01065-y (PMC8776838; doi:10.1038/s41396-021-01065-y)
Supplement: Supplementary file 1 — Supplemental Material [file 41396_2021_1065_MOESM1_ESM.pdf]

Supplementary Information

Materials and Methods

Preparation of PC+ media and iron substrates

PC+ was prepared by mixing 500 mL of artificial seawater (ASW) with 0.5 g casein, 0.5 g bacteriological peptone, 0.126 g NH_4Cl, and 1.8 g of glucose. The resulting solution was adjusted to pH 7.6, microwave sterilized, and stirred overnight with 7% w/v Chelex 100 resin (Bio-Rad, Hercules, CA, USA) to remove contaminating trace metals. This base media solution was filtered through a 0.2 μm filter to remove the Chelex resin and microwave sterilized an additional time. A trace metal master mix solution (4.0 x 10^{-5} M $ZnSO_4$, 2.3 x 10^{-4} M $MnCl_2$, 2.5 x 10^{-5} M $CoCl_2$, 1.0 x 10^{-5} M $CuSO_4$, 1.0 x 10^{-4} M Na_2MoO_4, 1.0 x 10^{-5} M Na_2SeO_3) was prepared in 0.1 M HCl. A vitamin mix solution (4.0 x 10^{-7} M cyanocobalamin, 2.0 x 10^{-6} M biotin, and 3.0 x 10^{-4} M thiamine HCl) was prepared in Milli-Q H_2O. Finally, a 0.735 M KH_2PO_4 solution in Milli-Q H_2O was prepared. The trace metal mix, vitamin mix, and KH_2PO_4 solutions were 0.2 μm filter sterilized and each diluted 1000-fold into aliquots of the base media immediately before inoculation with *A. macleodii* ATCC 27126. Individual iron stock solutions for experiments were prepared as described below and each added at a final total iron concentration of 5 x 10^{-6} M immediately prior to inoculation, unless otherwise noted.

$FeCl_3$ Control: A 5.0 x 10^{-3} M $FeCl_3 \bullet 6H_2O$ (Sigma-Aldrich, St. Louis, MO, USA) solution was prepared in 0.1 M HCl, 0.2 μm filter sterilized, and diluted 1000-fold into aliquots of the base PC+ media prior to inoculation with *A. macleodii* resulting in a final total iron concentration of 5 x 10^{-6} M.

Buffering Free Iron Concentrations with EDTA: A 5.0 x 10^{-2} M $Na_2EDTA \bullet 2H_2O$ (Sigma-Aldrich) solution was prepared in Milli-Q H_2O, adjusted to pH 8, 0.2 μm filter sterilized, and diluted either 1000-fold or 100-fold into aliquots of PC+ along with a 1000-fold dilution of the $FeCl_3$ stock solution described above prior to inoculation with *A. macleodii* resulting in a final total iron concentration of 5.0 x 10^{-6} M and final EDTA concentrations of 5.0 x 10^{-5} M and 5.0 x 10^{-4} M, respectively . Using the following equation, where L is EDTA and $K_{L,Fe'}^{cond}$ = $10^{7.3}$ (1), the estimated free iron (Fe′) concentrations in solution under these conditions are ~5.0 x 10^{-9} M and 0.5 x 10^{-9} M, respectively.

$$Fe' \cong \frac{[Fe_{total}]}{K_{L,Fe'}^{cond}[L]}$$

Synthesized Ferrihydrite Colloids: Ferrihydrite colloids were synthesized as described previously (2–4). Briefly, a 4.0 x 10^{-4} M $FeCl_3 \bullet 6H_2O$ (Sigma-Aldrich) solution was prepared in Milli-Q H_2O and allowed to polymerize at room temperature for 1 hour. This solution was then divided into two aliquots, one of which was incubated at 90°C for 5 minutes and then transferred to ice and the other of which was incubated at 90°C for 1 hour before transferring to ice. Solutions were stored overnight at 4°C before use and diluted 80-fold into aliquots of PC+ prior to inoculation with *A. macleodii* for a final total iron concentration of 5.0 x 10^{-6} M.

Arizona Test Dust: 0.0437 g of Arizona Test Dust (ATD) (A1 ultrafine test dust, 0-3 μm fraction) (Powder Technology Inc., Arden Hills, MN, USA) was suspended in 5 mL Milli-Q H_2O and microwave sterilized resulting in a total iron concentration of 5.0 x 10^{-3} M in the stock solution (5). The solution was stored overnight at 4°C before use and diluted 1000-fold into aliquots of PC+ prior to inoculation with *A. macleodii* resulting in a final total iron concentration of 5.0 x 10^{-6} M. Additional aliquots of ATD were subjected to a Berger leach (6) in order to

remove the most bioavailable fractions of iron. For the Berger leach, aliquots of ATD were leached in a 25% acetic acid, 0.02 M hydroxylamine hydrochloride solution with 10 minutes of heating at 90-95°C then allowed to cool to 30°C for an additional 1 hour and 50 minutes in the leach solution. The resulting Berger-leached ATD was dried and 0.0481 g was then suspended in 5 mL Milli-Q H_2O and microwave sterilized resulting in a total iron concentration of 5.0×10^{-3} M in the stock solution. The solution was stored overnight at 4°C before use and diluted 1000-fold into aliquots of PC+ prior to inoculation with *A. macleodii* resulting in a final total iron concentration of 5.0×10^{-6} M.

Suwanee River Humic Acid: A 1 mg/mL solution of Suwanee River Humic Acid standard (International Humic Substances Society, Denver, CO, USA) was prepared in Milli-Q H_2O resulting in a total iron concentration of 1.7×10^{-5} M in the stock solution (7). In order to remove the most labile fractions of iron from the solution, aliquots of the stock solution were extracted with a slurry of 5% (w/v) AG50W-X8 cation exchange resin (H^+ form, Bio-Rad) for either 30 minutes or overnight. Following extraction, solutions were passed through a 0.2 μm filter in order to remove the resin and sterilize. The unmodified solution was also 0.2 μm filter-sterilized and diluted 34-fold into aliquots of PC+ prior to inoculation with *A. macleodii* for a final iron concentration of 5.0×10^{-7} M (a 10-fold lower total iron concentration compared with other replete growth conditions due to limited substrate availability). The extracted aliquots of SRHA were also diluted 34-fold into aliquots of PC+ resulting in a lower (unknown) final iron concentration. Growth on varying concentrations of unmodified SRHA was also tested (5.0×10^{-8} M and 1.0×10^{-8} M final iron concentrations).

Ferrioxamine B: A 5.0×10^{-4} M $FeCl_3$ and 5.0×10^{-2} M desferrioxamine B (Sigma-Aldrich) solution was prepared in Milli-Q H_2O, 0.2 μm filter sterilized, and allowed to equilibrate

overnight. The equilibrated solution was diluted 100-fold into aliquots of PC+ prior to inoculation with *A. macleodii* resulting in a final total iron concentration of 5.0 x 10^{-6} M. Using the equation above where L is desferrioxamine B and $K_{L,Fe'}^{cond}$ = $10^{14.42}$ (8), the estimated Fe′ concentration under these conditions is ~3.8 x 10^{-17} M.

Ferric Citrate: A 5.0 x 10^{-4} M $FeCl_3$ and 5.0 x 10^{-2} M sodium citrate (Sigma-Aldrich) solution was prepared in Milli-Q H_2O, 0.2 μm filter sterilized, and allowed to equilibrate overnight. The equilibrated solution was diluted 100-fold into aliquots of PC+ prior to inoculation with *A. macleodii* resulting in a final total iron concentration of 5.0 x 10^{-6} M. Under these conditions (pH = 7.6 and a citrate:Fe ratio of 100:1), the dominant species in solution has been determined to be the monoiron dicitrate species $[Fe(citrate)_2]^{5-}$ (9).

Heme: A 5.0 x 10^{-4} M hemin chloride (Sigma-Aldrich) solution was prepared in 0.3 M NH_4OH, adjusted to pH 8, and 0.2 μm filter sterilized. The solution was then immediately diluted 100-fold into aliquots of PC+ prior to inoculation with *A. macleodii* resulting in a final total iron concentration of 5.0 x 10^{-6} M. This preparation of dissolved heme has been determined to have a maximum Fe′ concentration of 4 nM per 1 μM hemin chloride (10).

Cytochrome *c*: A 5.0 x 10^{-4} M cytochrome *c* (Sigma-Aldrich) solution was prepared in Milli-Q H_2O and 0.2 μm filter sterilized. The solution was then immediately diluted 100-fold into aliquots of PC+ prior to inoculation with *A. macleodii* resulting in a final total iron concentration of 5.0 x 10^{-6} M.

Phytoplankton Lysate: Phytoplankton lysate as an iron source was prepared from *Thalassiosira pseudonana* cultures as described in Hogle et al. (11). The resultant lysate was stored in liquid nitrogen until use and was diluted 2500-fold into aliquots of PC+ prior to inoculation with *A. macleodii*.

Siderophore characterization via solid phase extraction and LC-ESI-MS

Prior to preconcentration, the solid phase extraction (SPE) columns were activated using three column volumes of distilled methanol and were rinsed using acidified Milli-Q H_2O (pH 2; Optima HCl, Thermo Scientific, Waltham, MA, USA) followed by three column volume rinses with Milli-Q H_2O. Filtered culture extracts were preconcentrated onto a 6 mL Bond-Elut ENV SPE column (Agilent Technologies, Santa Clara, CA, USA) followed by a 500 mg Oasis HLB SPE column (Waters, Milford, MA, USA) to ensure the unknown siderophore was trapped by at least one of the columns. All steps were completed in the dark, and the columns were wrapped in aluminum foil during extraction. Filtered seawater collected on Gradients 1.0 was preconcentrated onto 6 mL Bond-Elut ENV SPE columns and stored at -20°C until further processing.

Following preconcentration, columns were eluted with 12 mL of distilled methanol into acid-cleaned 15 mL polyethylene tubes. Extracts were then dried down to approximately 500 µL over 4 hours at 35°C in a Speed-Vac concentrator coupled to a refrigerated vapor trap (Thermo Scientific). Concentrated extracts were then stored in the dark at 4°C until analysis.

Eluents were analyzed by first separating the compounds using reverse phase high pressure liquid chromatography (HPLC) on a PEEK ZORBAX-SB C18 column (0.5 x 150 mm, 5 µm particle size; custom column from Agilent Technologies). Compounds were detected by electrospray ionization mass spectrometry (ESI-MS) on a hybrid quadrupole Orbitrap (Q-Exactive HF; Thermo Scientific). Compounds were separated using a flow rate of 50 µL min^{-1} and a 20 minute gradient from 5 to 90% B, followed by a 5 minute gradient from 90 to 95% B,

and an isocratic gradient at 95% B for 5 minutes (solvent A: 5 mmol L^{-1} ammonium formate, solvent B: 5 mmol L^{-1} ammonium formate in distilled methanol). The ESI-MS contained a heated electrospray ionization source with a capillary temperature of 320°C, sheath gas of 16 and auxiliary gas equal to 3 and sweep gas equal to 1 (arbitrary units), spray voltage of 3.5 kV, auxiliary gas heater temperature of 90°C, and S-lens RF level of 65.0. MS^1 scans were collected in Full MS mode (200.0-2000.0 *m/z*) at 120,000 resolution in positive mode with a maximum injection time of 50. MS^2 spectra were collected using an isolation window of 1.0 *m/z* at 30,000 resolution targeting the most abundant masses and an inclusion list containing known siderophores (Chelomex) (12). The collision energy for MS^2 scans was set to 35 eV.

Data files were converted to an open source mzXML format using MSconvert (Proteowizard) and were processed using in-house R scripts (8). Known siderophores were searched based on MS^1 *m/z*, and fragmentation data was then further probed from putatively identified compounds and compared to literature MS^2 spectra.

Identifying petrobactin biosynthetic pathways in sequenced bacterial isolates

A search for homologous pathways for petrobactin biosynthesis was conducted with genomes deposited in the European Nucleotide Archive (ENA) using the Enzyme Similarity and Genome Neighborhood tools of the Enzyme Function Initiative (13,14). The InterPro domain IPR013022 was used to first identify sequences homologous to *asbF*, the gene encoding 3-dehydroshikimate dehydratase found within the petrobactin biosynthetic pathway (Figure 1, main text). The genome neighborhoods (+/- 10 genes) of the detected 3-dehydroshikimate dehydratase homologs

were then retrieved and filtered for those that contained homologs to NIS-type siderophore synthetases (pfam04183).

Results

Identifying petrobactin biosynthetic pathways in sequenced bacterial isolates

A search for biosynthetic pathways homologous to those from known petrobactin producers was conducted in genomes from isolated strains deposited in the ENA and returned 602 strains with putative petrobactin biosynthetic pathways (Figure S7, Dataset S1). Over 400 of these positive hits consisted of strains from the *Bacillales* order. The remainder of identified strains fall within the *Alphaproteobacteria* and *Gammaproteobacteria* classes. Within these groups, positive hits for *Alteromonas*, *Marinobacter*, and *Rhodopseudomonas* strains were detected as expected based on known petrobactin production (15,16). Additional taxa with putative petrobactin biosynthetic capabilities include additional species from the order *Rhizobiales* as well as *Rhodobacterales*, *Rhodospirillales*, *Vibrionales*, *Aeromonadales*, *Oceanospirillales*, and *Cellvibrionales*. A number of these isolated strains, such as *Alteromonas macleodii* English Channel 673, are among the most prevalent species in the Tara Oceans dataset (17).

References

1. Maldonado MT, Strzepek RF, Sander S, Boyd PW. Acquisition of iron bound to strong organic complexes, with different Fe binding groups and photochemical reactivities, by plankton communities in Fe-limited subantarctic waters. *Global Biogeochem Cycles*. 2005; 19: GB4S23.
2. Wells ML, Mayer LM, Guillard RRL. A chemical method for estimating the availability

of iron to phytoplankton in seawater. *Mar Chem*. 1991; 33(1–2): 23–40.

3. Wells ML, Zorkin NG, Lewis AG. The role of colloid chemistry in providing a source of iron to phytoplankton. *J Mar Res.* 1983; 41(4): 731–46.

4. Barbeau K, Moffett JW, Caron DA, Croot PL, Erdner DL. Role of protozoan grazing in relieving iron limitation of phytoplankton. *Nature*. 1996; 380(6569): 61–4.

5. Perron MMG, Strzelec M, Gault-Ringold M, Proemse BC, Boyd PW, Bowie AR. Assessment of leaching protocols to determine the solubility of trace metals in aerosols. *Talanta.* 2020; 208: 120377.

6. Berger CJM, Lippiatt SM, Lawrence MG, Bruland KW. Application of a chemical leach technique for estimating labile particulate aluminum, iron, and manganese in the Columbia River plume and coastal waters off Oregon and Washington. J *Geophys Res.* 2008; 113: C00B01.

7. Laglera LM, van den Berg CMGG. Evidence for geochemical control of iron by humic substances in seawater. *Limnol Oceanogr*. 2009; 54(2): 610–9.

8. Bundy RM, Boiteau RM, McLean C, Turk-Kubo KA, McIlvin MR, Saito MA, et al. Distinct Siderophores Contribute to Iron Cycling in the Mesopelagic at Station ALOHA. *Front Mar Sci*. 2018; 5: 61.

9. Silva AMN, Kong X, Parkin MC, Cammack R, Hider RC. Iron(iii) citrate speciation in aqueous solution. *Dalt Trans*. 2009; (40): 8616–25.

10. Rao AU, Carta LK, Lesuisse E, Hamza I. Lack of heme synthesis in a free-living eukaryote. *Proc Natl Acad Sci U S A*. 2005; 102(12): 4270–5.

11. Hogle SL, Brahamsha B, Barbeau KA. Direct Heme Uptake by Phytoplankton-Associated Roseobacter Bacteria. *mSystems*. 2017; 2(1): e00124-16.

12. Baars O, Morel FMM, Perlman DH. ChelomEx: Isotope-assisted discovery of metal chelates in complex media using high-resolution LC-MS. *Anal Chem*. 2014; 86(22): 11298–305.

13. Gerlt JA, Bouvier JT, Davidson DB, Imker HJ, Sadkhin B, Slater DR, et al. Enzyme Function Initiative-Enzyme Similarity Tool (EFI-EST): A web tool for generating protein sequence similarity networks. *Biochim Biophys Acta.* 2015; 1854(8): 1019–37.

14. Gerlt JA. Genomic Enzymology: Web Tools for Leveraging Protein Family Sequence−Function Space and Genome Context to Discover Novel Functions. *Biochemistry*. 2017; 56: 4293–308.

15. Barbeau K, Zhang G, Live DH, Butler A. Petrobactin, a photoreactive siderophore produced by the oil-degrading marine bacterium Marinobacter hydrocarbonoclasticus. J *Am Chem Soc*. 2002; 124(3): 378–9.

16. Baars O, Morel FMM, Zhang X. The purple non-sulfur bacterium Rhodopseudomonas palustris produces novel petrobactin-related siderophores under aerobic and anaerobic conditions. *Environ Microbiol*. 2018; 20(5): 1667–76.

17. Nayfach S, Rodriguez-Mueller B, Garud N, Pollard KS. An integrated metagenomics pipeline for strain profiling reveals novel patterns of bacterial transmission and biogeography. *Genome Res*. 2016; 26(11): 1612–25.

Table S1 Gibson assembly

Gibson Fragment	Forward Primer (5' to 3')	Reverse Primer (5' to 3')	Source DNA (Reference, Main Text)	Fragment Length (bp)
**Fragment_1** ***sacB*****,** ***oriV*****,** ***oriT***	AAAACGAAAAGCCTGA TAATACTCCACCTGCAG GTCGACTCTAGCTAGAG	CCTTCTTGAACAAGTTG ATCGGCACATCTTCCGC TGCATAACCCTGCTTC	pRED16 (41)	4942
**Fragment_2 Homologous Region 1**	GAAGCAGGGTTATGCA GCGGAAGATGTGCCGA TCAACTTGTTCAAGAAG G	TCTCATGAGCGGATACA TATTTGAAAAAAACAGT AACGTAGGTTATCCCC	*Alteromonas macleodii* ATCC 27126 genomic DNA	1050
**Fragment_3** **Km**^{R}	GGGGATAACCTACGTTA CTGTTTTTTTCAAATAT GTATCCGCTCATGAGA	AATTCGGTTCGTTCAGC ACACTGTCAGACCAAGT TTACTCATATATACT	pRED2 (41)	1101
**Fragment_4 Homologous Region 2**	AAAATGAAGTTTTAAAT CAATCTAATGTGCTGAA CGAACCGAATTATCAA	CTCTAGCTAGAGTCGAC CTGCAGGTGGAGTATTA TCAGGCTTTTCGTTTT	*Alteromonas macleodii* ATCC 27126 genomic DNA	1050

Table S2 List of primers used for PCR verification reactions

Primer	Sequence (5' to 3')	Description
HR1_F	GTGCCGATCAACTTG TTCAAGAAGG	Used to confirm correct plasmid orientation following Gibson assembly of pLEM01
Kan_R	CTGTCAGACCAAGTT TACTCATATATACT	Used to confirm correct plasmid orientation following Gibson assembly of pLEM01
Kan_F	TTCAAATATGTATCC GCTCATGAGA	Used to confirm correct plasmid orientation following Gibson assembly of pLEM01
HR2_R	GGAGTATTATCAGGC TTTTCGTTTT	Used to confirm correct plasmid orientation following Gibson assembly of pLEM01
insertion_F	GTGCCGATCAACTTG TTCAAGAAGG	Used to confirm double crossover insertion in *ΔasbB*::km^r mutant strain
insertion_R	GGAGTATTATCAGGC TTTTCGTTTT	Used to confirm double crossover insertion in *ΔasbB*::km^r mutant strain
deletion_F	ATCCGTGGCAATATG ACAAGCTAAA	Used to confirm deletion in *ΔasbB*::km^r mutant strain
deletion_R	AAAGATAACACTAAG GCTTGGGGAT	Used to confirm deletion in *ΔasbB*::km^r mutant strain

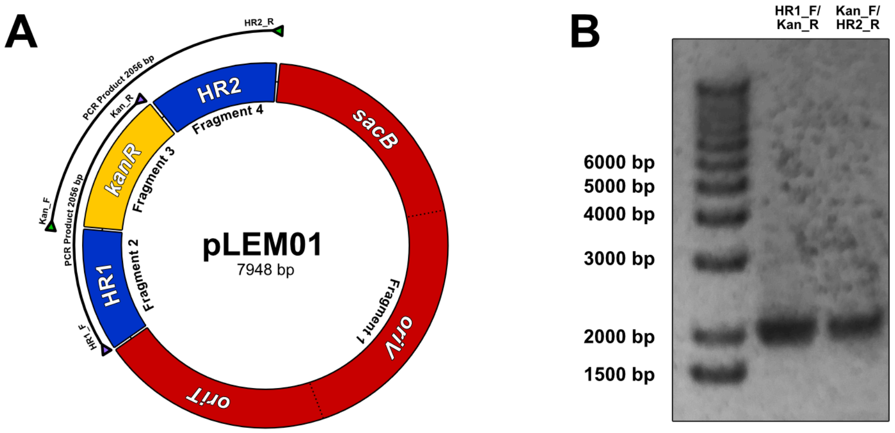

Figure S1 (A) Representation of pLEM01 following Gibson assembly. Individually colored Gibson fragments (1- 4) are labeled with corresponding genomic features as described in Table S1. Primers used for verification of correct assembly are depicted as triangles and labeled according to Table S2 with the expected length of the PCR amplified product also displayed. **(B)** Gel image depicting PCR amplified products with corresponding primers. Leftmost lane contains 1kb plus DNA ladder (Invitrogen).

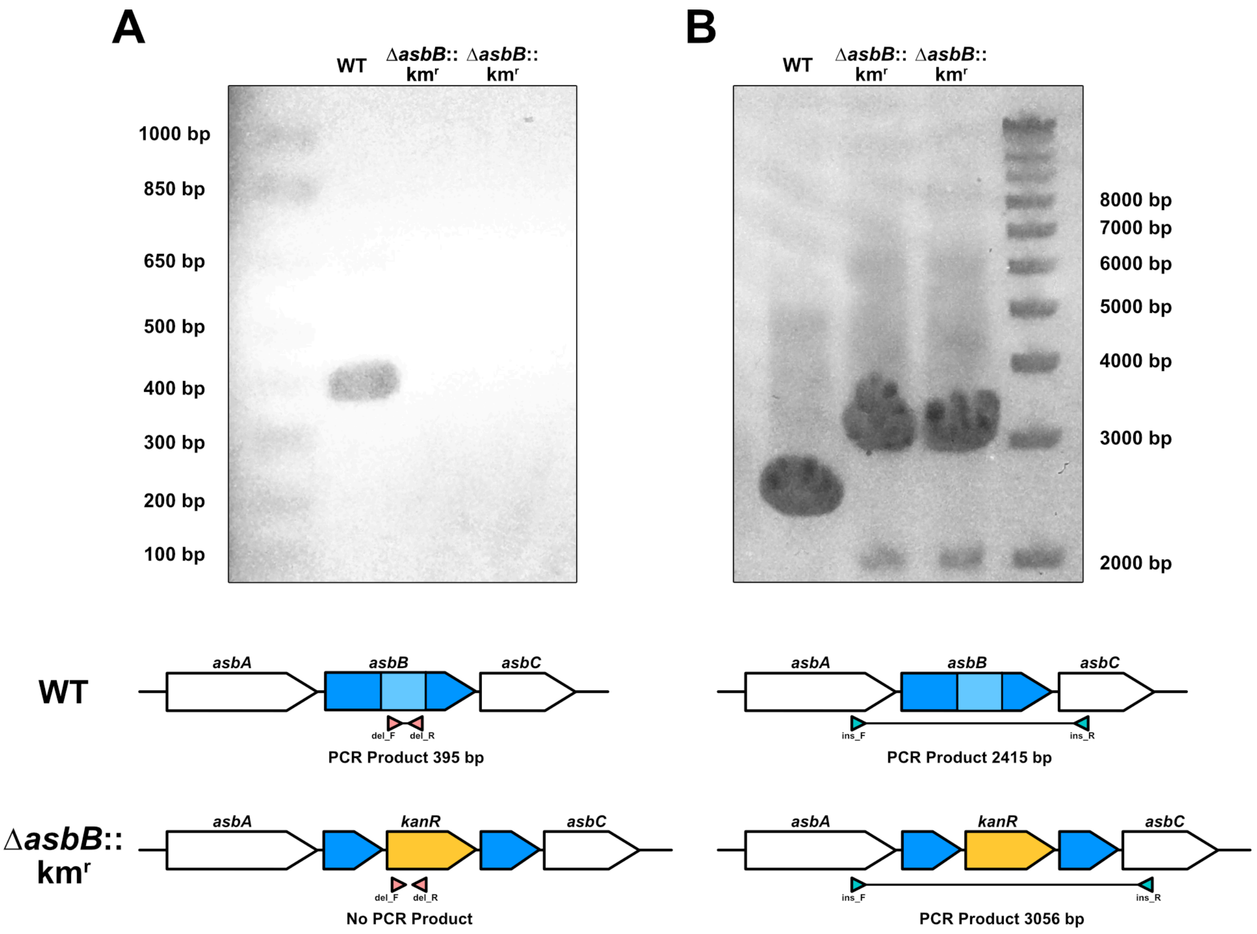

Figure S2 (A) PCR verification of the anticipated 415 bp deletion from *asbB* following homologous recombination. Gel image depicts the PCR amplified product from the WT strain using the del_F and del_R primers (Table S2). As expected, no PCR product was detected using these primers in the Δ*asbB*::km^r strain due to insertion of the kanamycin resistance cassette. Leftmost lane contains 1kb plus DNA ladder (Invitrogen). **(B)** PCR verification of the anticipated insertion of the kanamycin resistance cassette following homologous recombination. Gel image depicts the PCR amplified products from the WT and Δ*asbB*::km^r strains using the ins_F and ins_R primers (Table S2) confirming the expected 641 bp difference between amplified fragments due to the insertion of *kanR* (1056 bp) and the concurrent 415 bp deletion. Rightmost lane contains 1kb plus DNA ladder (Invitrogen).

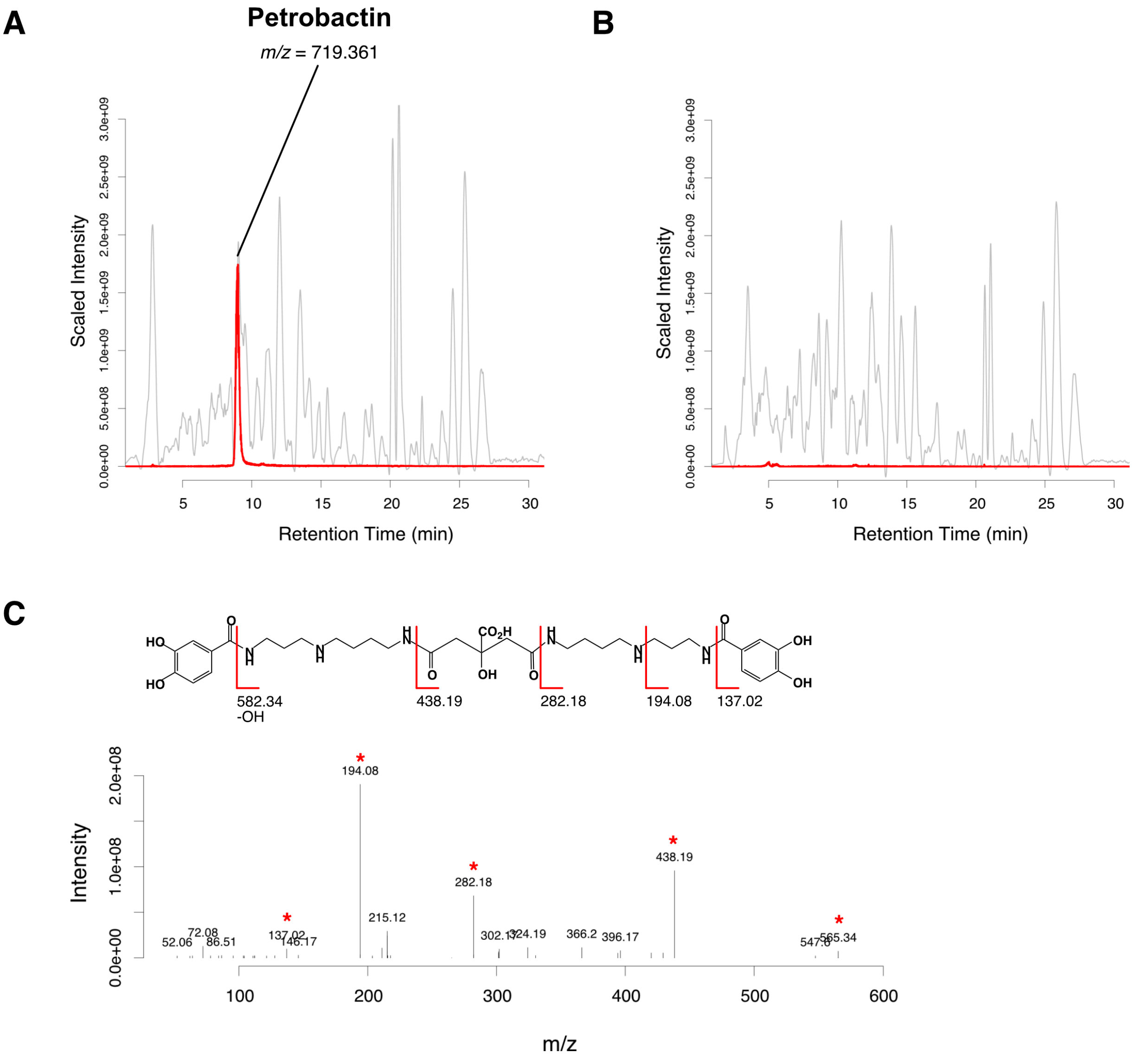

Figure S3 (A) The total ion chromatogram of the supernatant extracts from *A. macleodii* ATCC 27126 WT grown under iron limitation is displayed in the gray trace with the extracted peak for the expected *m/z* of petrobactin (719.361) overlain in red at a scaled intensity. **(B)** The total ion chromatogram of the supernatant extracts from *A. macleodii* ATCC 27126 Δ*asbB*::km^r grown under iron limitation is displayed in the gray trace. The extracted chromatogram at 719.361 *m/z* in red displays the absence of a petrobactin peak, confirming the knockout phenotype. **(C)** The MS^2 spectra collected at 719.361 *m/z* from the WT extract. The petrobactin structure is shown for reference and annotated with expected fragmentation. Corresponding fragment peaks detected in the spectra are noted with red asterisks.

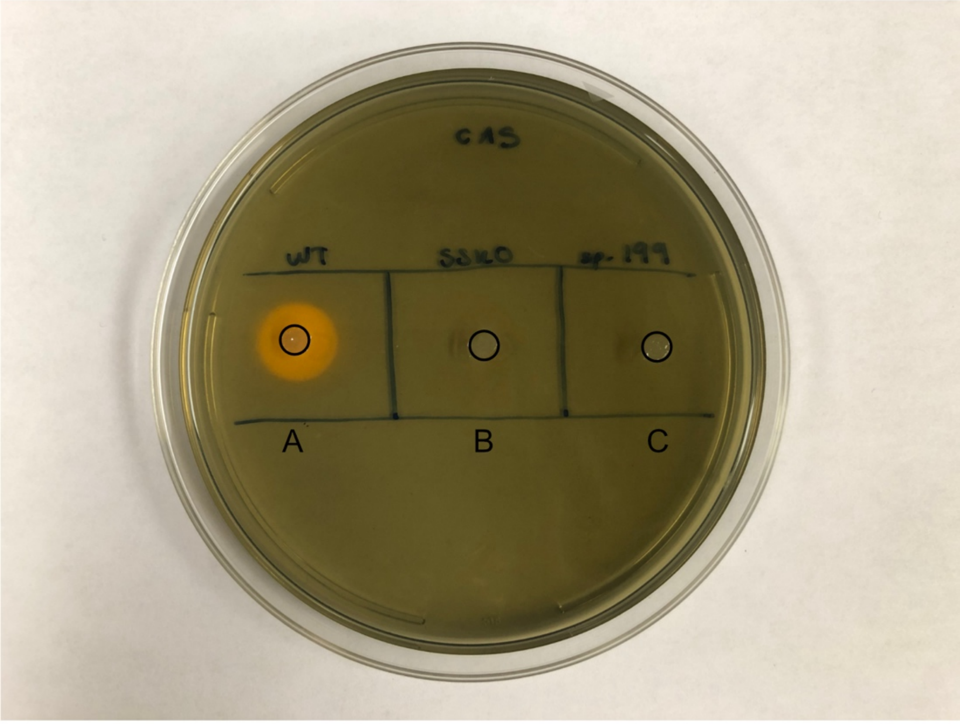

Figure S4 (A) CAS assay detecting siderophore production in *A. macleodii* ATCC 27126 WT strain as indicated by orange halo extending beyond cells outlined in black. **(B)** No siderophore production was detected in the Δ*asbB*::km^r strain confirming the knockout phenotype. **(C)** An additional *Alteromonas* strain, A. *sp.* 199, was used as a negative control as no siderophore biosynthetic pathway has been detected within its genome.

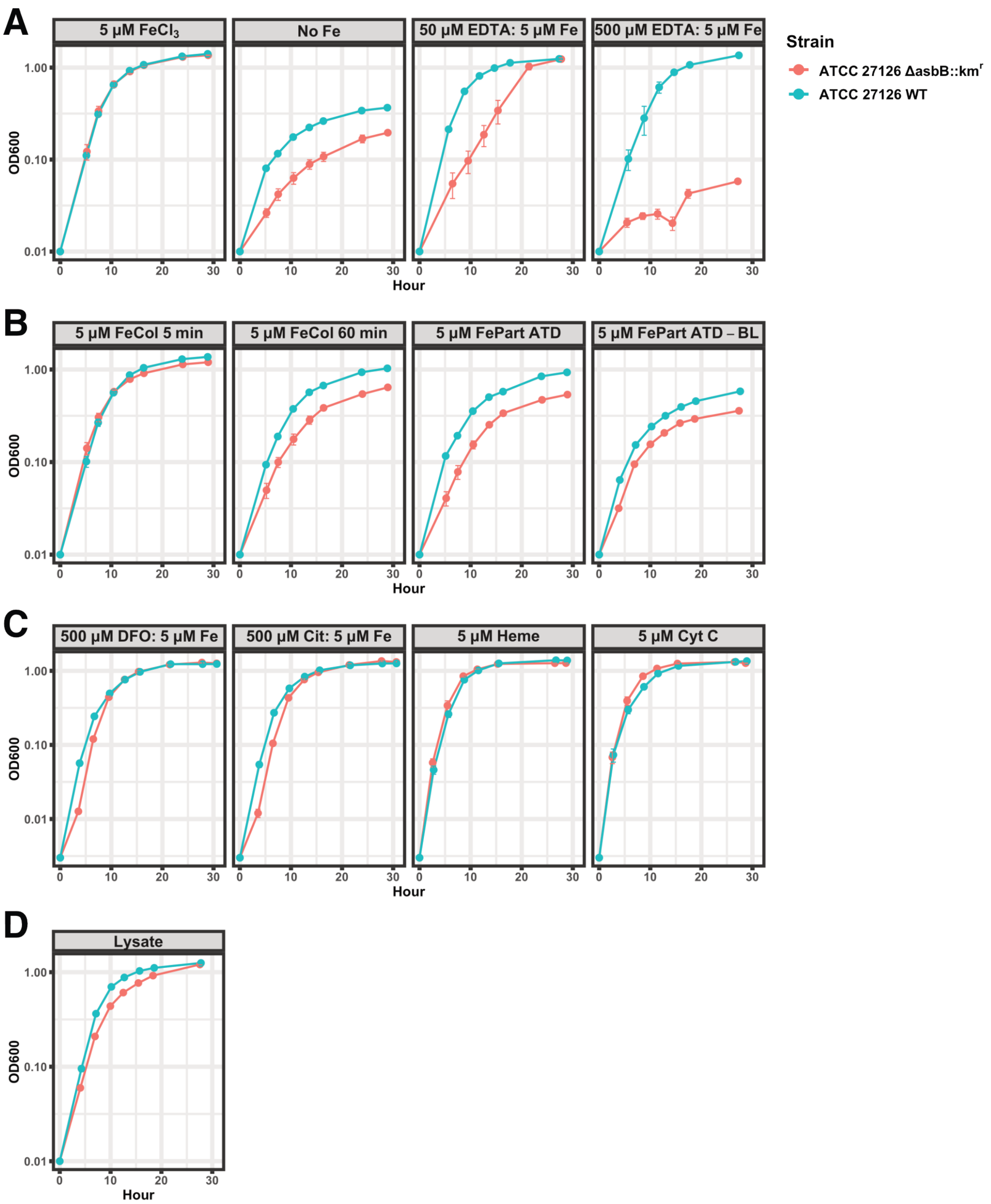
A
B
C
D
5 μM FeCl3
No Fe
50 μM EDTA: 5 μM Fe
500 μM EDTA: 5 μM Fe
5 μM FeCol 5 min
5 μM FeCol 60 min
5 μM FePart ATD
5 μM FePart ATD – BL
500 μM DFO: 5 μM Fe
500 μM Cit: 5 μM Fe
5 μM Heme
5 μM Cyt C
Lysate
OD600
Hour
1.00
0.10
0.01
0
10
20
30
Strain
ATCC 27126 ΔasbB::kmr
ATCC 27126 WT

Figure S5 Growth curves of *A. macleodii* ATCC 27126 WT and Δ*asbB*::km^r strains on various sources of iron. The y-axis (log_{10} scale) displays growth as optical density measurements at 600 nm versus time in hours on the x-axis. Error bars represent the standard deviation of measurements from biological triplicates. **(A)** Iron controls and EDTA treatments **(B)** Colloidal and particulate mineral iron sources, 5 μM FeCol (5 min): Fe colloids synthesized with 5 minutes of heating and added at a total iron concentration of 5 μM, 5 μM FeCol (60 min): Fe colloids synthesized with 60 minutes of heating and added at a total iron concentration of 5 μM, 5 μM FePart ATD: particulate iron added as Arizona Test Dust at a total iron concentration of 5 μM, 5 μM FePart BL-ATD: particulate iron added as Berger-leached Arizona Test Dust at a total iron concentration of 5 μM **(C)** Biogenic sources of chelated iron each added at a total iron concentration of 5μM, DFO: desferrioxamine B, Cit: citrate, Cyt c: cytochrome *c* **(D)** *T. pseudonana* lysate added as the sole iron source

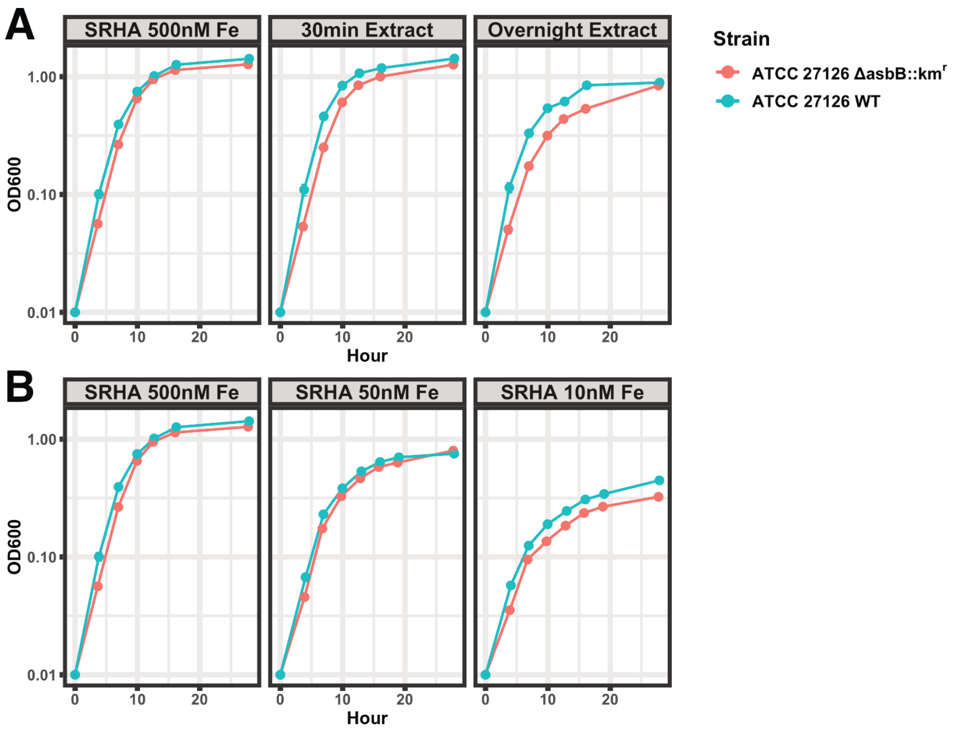

Figure S6 Growth of *A. macleodii* ATCC 27126 WT and Δ*asbB*::km^r strains on Suwanee River Humic Acid (SRHA) as the sole iron source. The y-axis (log_{10} scale) displays growth as optical density measurements at 600 nm versus time in hours on the x-axis. Error bars represent the standard deviation of measurements from biological triplicates. **(A)** SRHA 500 nM Fe: unmodified SRHA added at a final iron concentration of 500 nM, 30 min Extract: SRHA extracted for 30 minutes with AG50W-X8 cation exchange resin and added at same volume as unmodified SRHA to final cultures, Overnight Extract: SRHA extracted overnight with AG50W-X8 cation exchange resin and added at same volume as unmodified SRHA to final cultures **(B)** SRHA 500 nM Fe: unmodified SRHA added at a final iron concentration of 500 nM, SRHA 50 nM Fe: unmodified SRHA added at a final iron concentration of 50 nM, SRHA 10 nM Fe: unmodified SRHA added at a final iron concentration of 10 nM

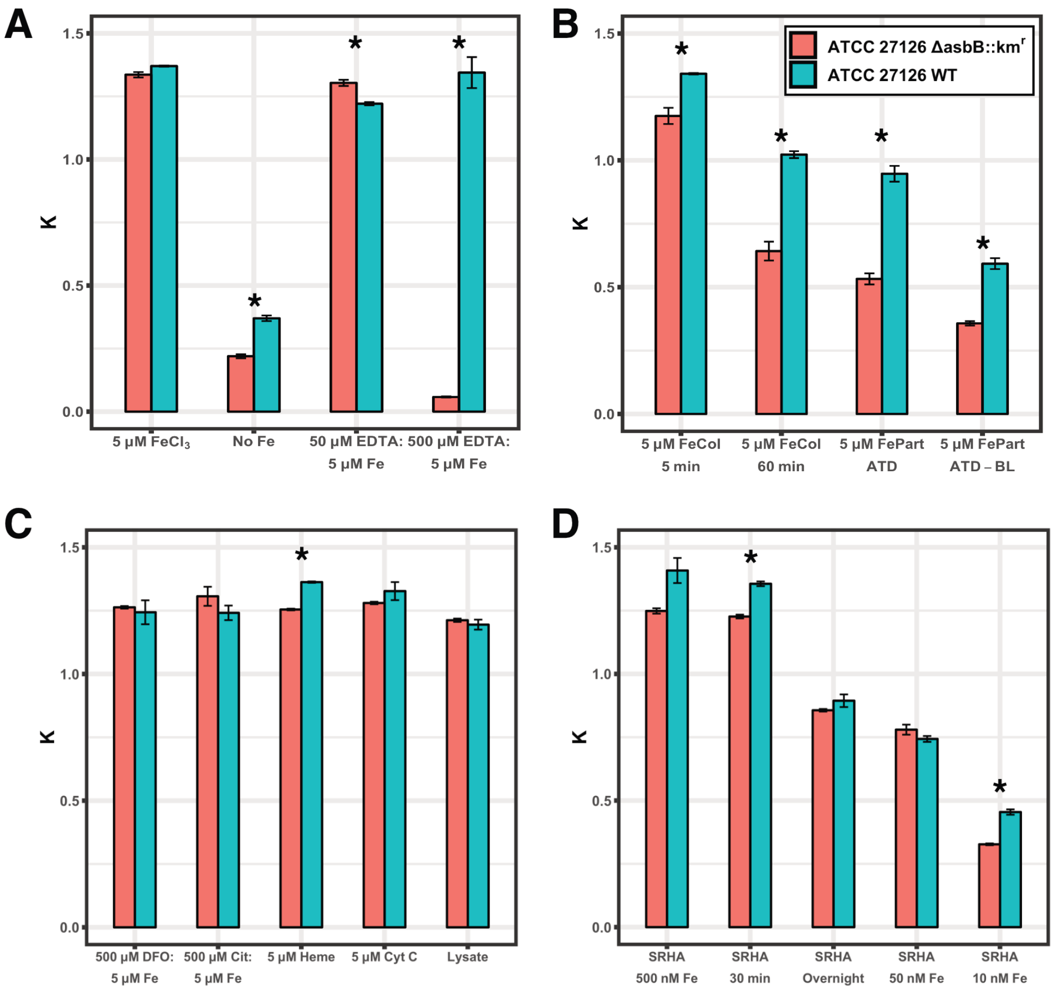

Figure S7 Carrying capacity (K) of the *A. macleodii* ATCC 27126 WT and Δ*asbB*::km^r strains on tested iron sources. Conditions marked with an asterisk indicate a statistically significant difference between the WT and Δ*asbB*::km^r strains (independent, two-tailed T-test, $p < 0.05$). Error bars represent the standard deviation of measurements from biological triplicates **(A)** Iron controls and EDTA treatments **(B)** Colloidal and particulate mineral iron sources, 5 µM FeCol (5 min): Fe colloids synthesized with 5 minutes of heating and added at a total iron concentration of 5 µM, 5 µM FeCol (60 min): Fe colloids synthesized with 60 minutes of heating and added at a total iron concentration of 5 µM, 5 µM FePart ATD: particulate iron added as Arizona Test Dust at a total iron concentration of 5 µM, 5 µM FePart BL-ATD: particulate iron added as Berger-leached Arizona Test Dust at a total iron concentration of 5 µM **(C)** Biogenic sources of chelated iron each added at a total iron concentration of 5µM, DFO: desferrioxamine B, Cit: citrate, Cyt c: cytochrome *c*, Lysate: *T. pseudonana* lysate added as the sole iron source **(D)** SRHA treatments, SRHA 500 nM Fe: unmodified SRHA added at a final iron concentration of 500 nM, SRHA(30 min: SRHA extracted for 30 minutes with AG50W-X8 cation exchange resin and added at same volume as unmodified SRHA to final cultures, SRHA Overnight: SRHA extracted overnight with AG50W-X8 cation exchange resin and added at same volume as unmodified SRHA to final cultures, SRHA 50 nM Fe: unmodified SRHA added at a final iron concentration of 50 nM, SRHA 10 nM Fe: unmodified SRHA added at a final iron concentration of 10 nM

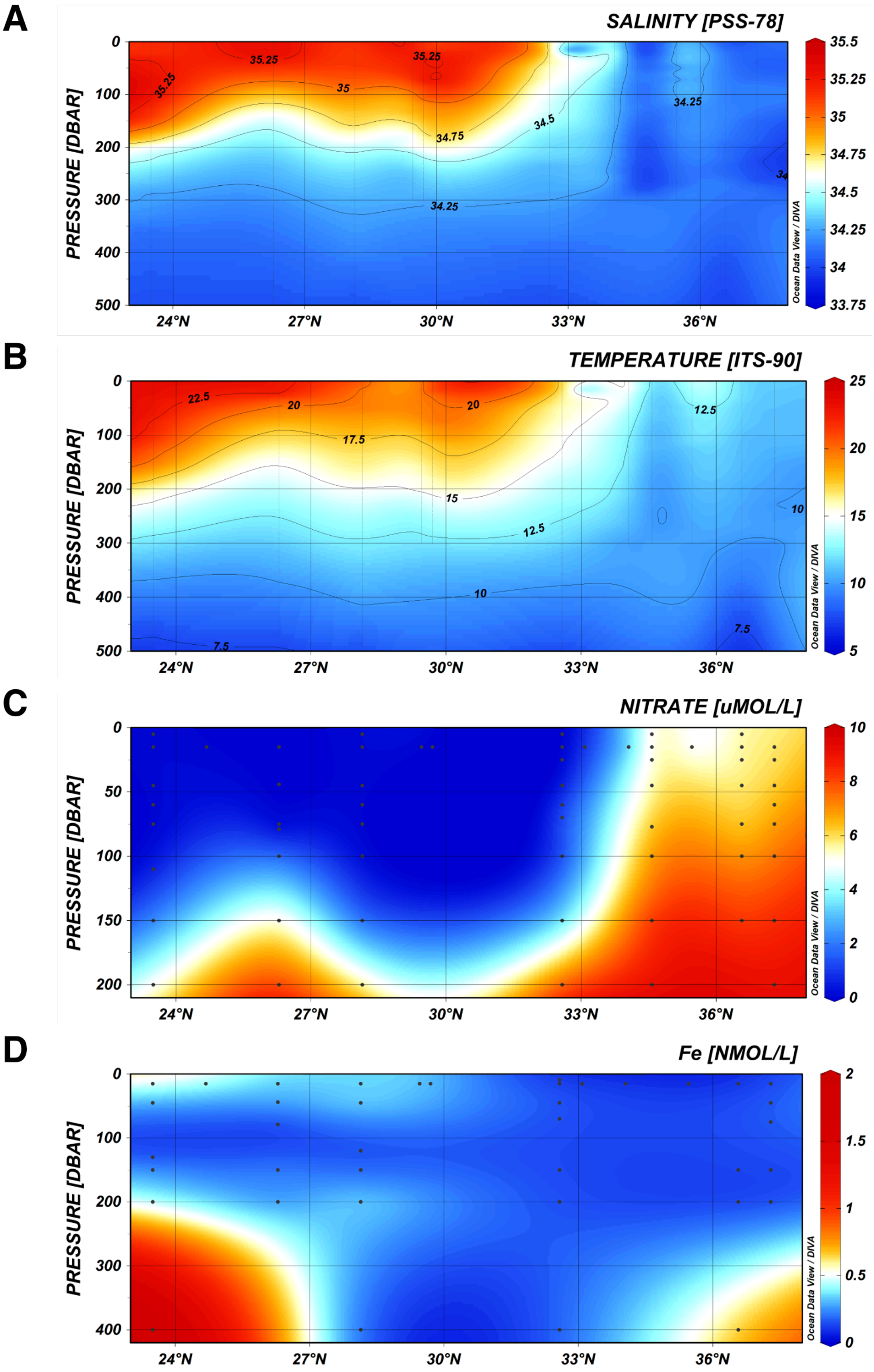

A
SALINITY [PSS-78]
B
TEMPERATURE [ITS-90]
C
NITRATE [uMOL/L]
D
Fe [NMOL/L]
PRESSURE [DBAR]
24°N
27°N
30°N
33°N
36°N
Ocean Data View / DIVA

Figure S8 Physiochemical data collected on the Gradients 1.0 cruise along 158°W between 23 and 38°N during April 2016. **(A)** Salinity profiles from the surface to 500 m. **(B)** Temperature (°C) profiles from the surface to 500 m. **(C)** Nitrate concentration (µmol/L) profiles from the surface to 200 m. **(D)** Dissolved iron concentration (nmol/L) profiles from the surface to 400 m.

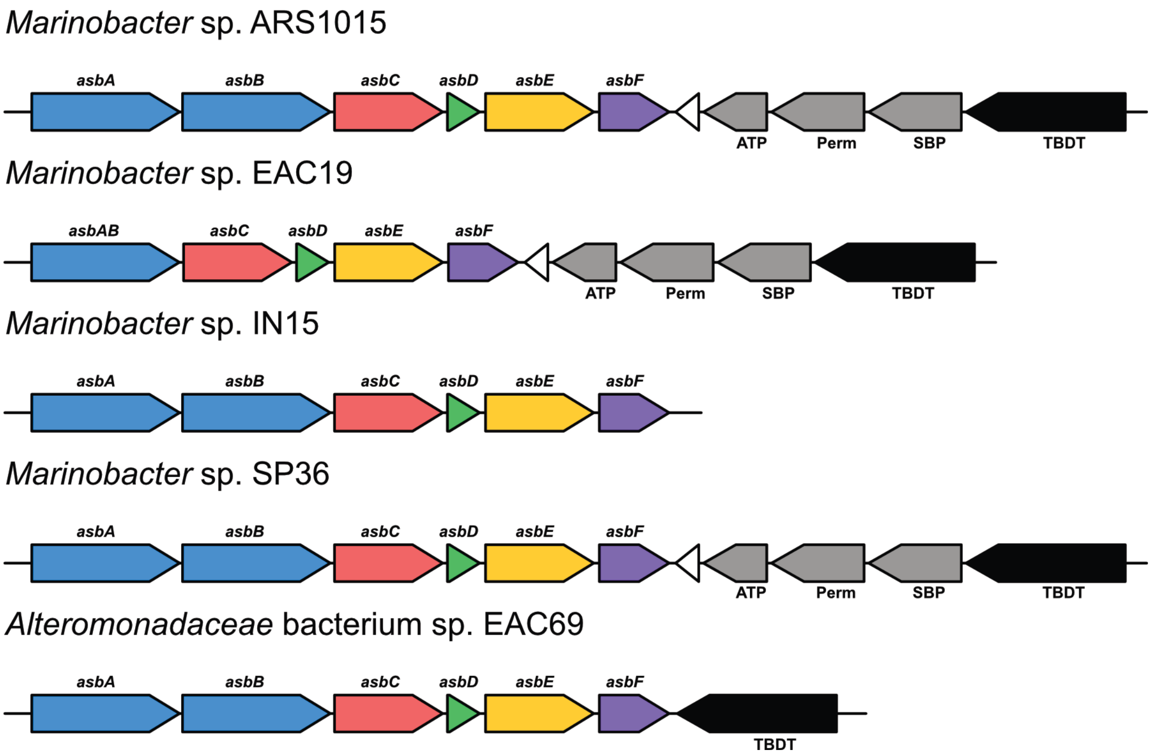

Figure S9 Biosynthetic gene clusters identified from *Tara* Ocean MAGs that are homologous to the petrobactin biosynthetic pathway as characterized in *B. antrhacis*. Genes labeled and colored according to homology as in Figure 1 in main text. Co-located genes encoding putative petrobactin transport systems are also depicted (black and gray) with hypothetical genes displayed in white. TBDT: tonB dependent transporter, SBP: solute binding protein, Perm: permease, ATP: ATP-binding protein

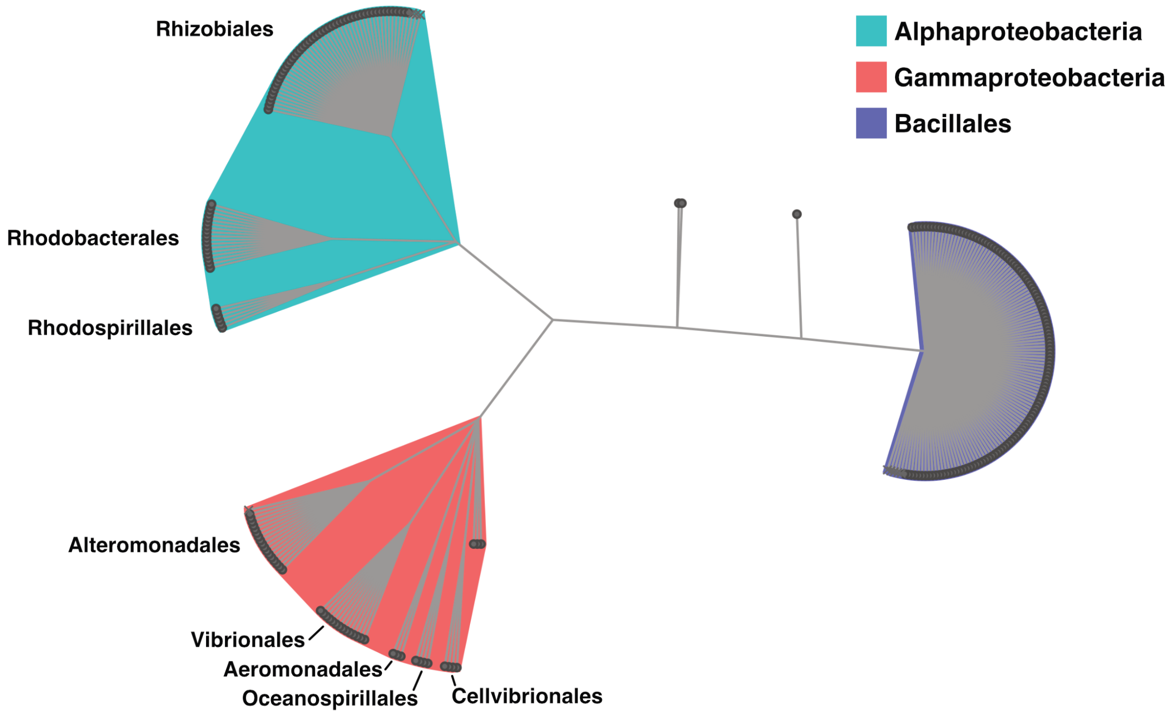

Figure S10 Taxonomic distribution of sequenced isolates from the European Nucleotide Archive with putative petrobactin-like biosynthetic gene clusters. Nodes displayed at species level.

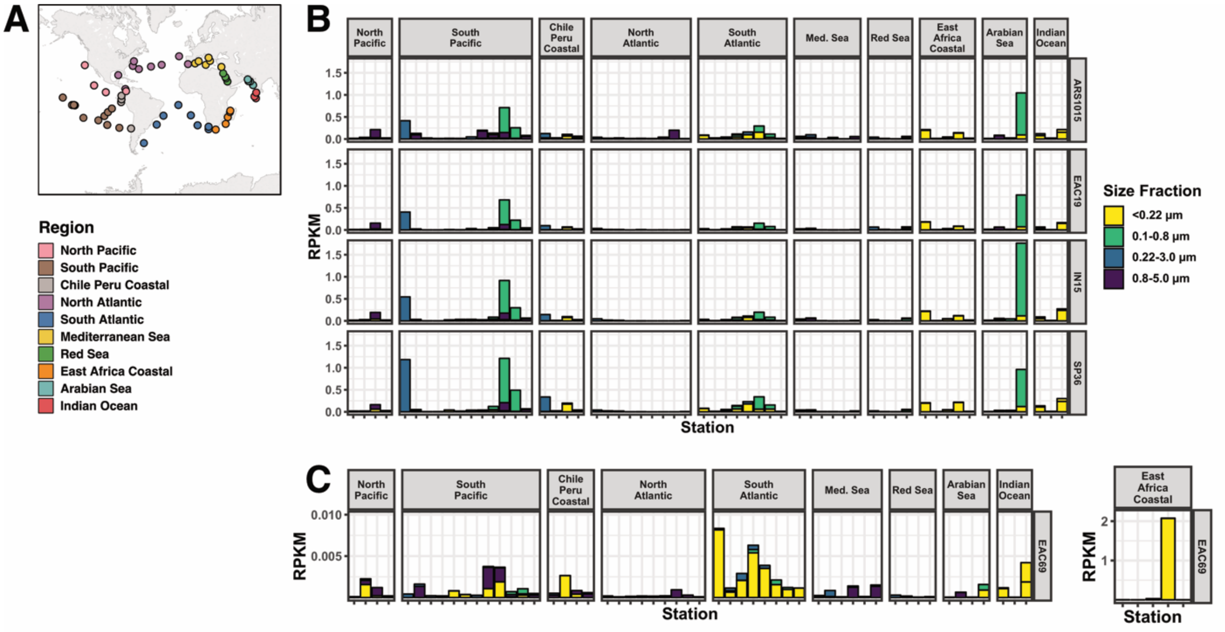

Figure S11 Distribution of reads mapped to MAGs containing a complete petrobactin-like biosynthetic gene cluster in surface samples from the *Tara* Oceans dataset. **(A)** Station map of *Tara* Oceans sampling locations colored according to region. **(B)** Abundance of reads (RPKM normalized) mapped to four *Marinobacter* MAGs at each surface station across the *Tara* Oceans dataset. Samples grouped according to oceanic region as defined in panel A and colored by corresponding sample size fraction. **(C)** Abundance of reads (RPKM normalized) mapped to an *Alteromonadaceae* MAG at each surface station across the *Tara* Oceans dataset. Samples grouped according to oceanic region as defined in panel A and colored by corresponding sample size fraction. Stations from the East Africa Coastal region shown on a separate scale for clarity.

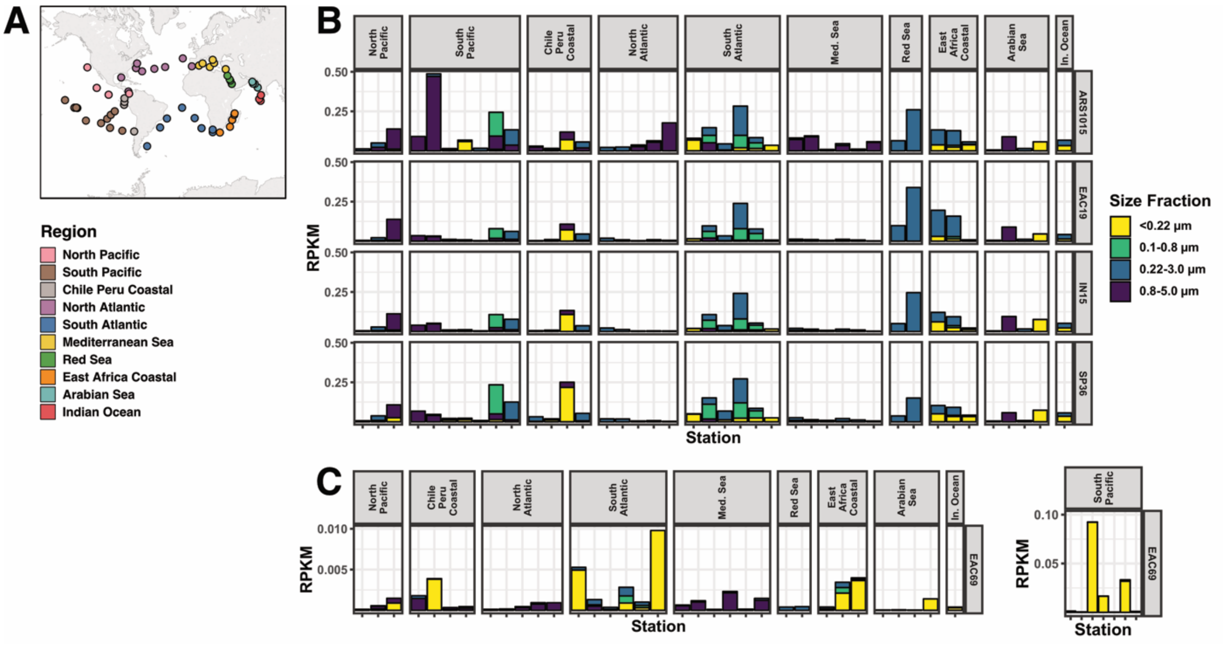

Figure S12 Distribution of reads mapped to MAGs containing a complete petrobactin-like biosynthetic gene cluster in DCM samples from the *Tara* Oceans dataset. **(A)** Station map of *Tara* Oceans sampling locations colored according to region. **(B)** Abundance of reads (RPKM normalized) mapped to four *Marinobacter* MAGs at each DCM station across the *Tara* Oceans dataset. Samples grouped according to oceanic region as defined in panel A and colored by corresponding sample size fraction. **(C)** Abundance of reads (RPKM normalized) mapped to an *Alteromonadaceae* MAG at each DCM station across the *Tara* Oceans dataset. Samples grouped according to oceanic region as defined in panel A and colored by corresponding sample size fraction. Stations from the South Pacific region shown on a separate scale for clarity.
